# Photodynamic Antifungal Activity of Hypocrellin A Against *Candida albicans*

**DOI:** 10.3389/fmicb.2019.01810

**Published:** 2019-08-06

**Authors:** Yijia Yang, Chenglu Wang, Yingzhi Zhuge, Jian Zhang, Ke Xu, Qilu Zhang, Haijuan Zhang, Haiyan Chen, Maoping Chu, Chang Jia

**Affiliations:** ^1^Pediatric Research Institute, The Second Affiliated Hospital and Yuying Children’s Hospital, Wenzhou Medical University, Wenzhou, China; ^2^Children’s Heart Center, Institute of Cardiovascular Development and Translational Medicine, The Second Affiliated Hospital and Yuying Children’s Hospital, Wenzhou Medical University, Wenzhou, China; ^3^The Second Clinical Medical College, Wenzhou Medical University, Wenzhou, China; ^4^The Institute of Life Sciences, Wenzhou University, Wenzhou, China; ^5^School of Pharmaceutical Sciences, Wenzhou Medical University, Wenzhou, China

**Keywords:** hypocrellin A, *Candida albicans*, cell membrane, apoptosis, skin infections

## Abstract

Many studies have reported that hypocrellin A (HA) exhibits effective antimicrobial activities with proper irradiation. However, its antifungal activity and the involved mechanism have not been fully defined. In this study, HA-mediated cytotoxicity in *Candida albicans* cells was evaluated after antimicrobial photodynamic therapy (aPDT). The results showed that 1.0 μg/ml HA significantly decreased the survival rate of *C. albicans* cells with light illumination. Moreover, the ROS levels were also remarkably elevated by HA. Further study found that HA combined with illumination led to cell membrane potential depolarization and cell membrane integrity damage. To investigate the form of cell death, a series of apoptosis-related parameters, including mitochondrial transmembrane potential, metacaspase activity, DNA fragmentation, nuclear condensation, and cytosolic and mitochondrial calcium, were analyzed. Data showed that all the above mentioned apoptosis hallmarks were affected after treatment with HA, indicating that HA induced *C. albicans* cell apoptosis. Finally, HA-mediated aPDT was demonstrated to be low-toxic and effective in treating cutaneous *C. albicans* infections. This study highlights the antifungal effect and mechanism of HA-mediated aPDT against *C. albicans* and provides a promising photodynamic antifungal candidate for *C. albicans* skin infections.

## Introduction

*Candida albicans*, as a kind of major opportunistic fungal pathogens, can cause both superficial and life-threatening infections in immunocompromised hosts. Although rarely life-threatening, these superficial fungal skin infections can cause debilitating effects on a person’s quality of life and may become invasive in some circumstances. Topical antifungal drugs, such as clotrimazole, are the most commonly utilized options for treating cutaneous *C. albicans* infections. However, the resistance of *C. albicans* to clotrimazole has been reported (Martel et al., 2010), and the incidence of resistance to antifungal drugs may be increasing (Fischer et al., 2010). Therefore, it is imperative to explore alternative antifungal agents or strategies that are effective against *C. albicans* skin infections.

Antimicrobial photodynamic therapy (aPDT) is a recently developed therapeutic option, based on utilizing non-toxic photosensitizer (PS) and visible light to induce the production of reactive oxygen species (ROS) that can kill the microbial pathogens that have bound with PS (Dai et al., 2011; Hamblin, 2016). Numerous studies have demonstrated that aPDT was highly effective in the *in vitro* and *in vivo* inactivation of fungi (Mima et al., 2010; Dai et al., 2011; Dovigo et al., 2011; Pereira et al., 2011; Lívia Nordi et al., 2013). Moreover, it is regarded that it is difficult for fungi to develop the resistance to aPDT since this strategy-mediated killing is a non-specific and multi-target process, which is also one advantage of aPDT over conventional antifungals (Mima et al., 2012; Hamblin, 2016). Although microbial cells, including fungi, possess several mechanisms that can detoxify ROS and overcome toxicity of photooxidative stress generated by aPDT, still many papers report that aPDT has (so far) not been shown to produce resistance in bacteria (Su et al., 2011; Maisch, 2015; Hamblin, 2016) and fungi (Baltazar et al., 2015; Hamblin, 2016; Suzuki et al., 2017; Paramanantham et al., 2018). Thereby, antifungal aPDT will become more and more important in the future as antifungal resistance is expected to continue to be increasing.

Hypocrellin A (HA), as a type of PS, is a lipid-soluble perylenequinone pigment extracted from the fungi of *Hypocrella bambusae* (He and Jiang, 2000). This compound, as orally token folk medicine, has been used to treat gastric diseases and rheumatoid arthritis for centuries (Zhenjun and Lown, 2010). Several studies have reported that HA exhibits effective anticancer, antiviral and antimicrobial activities (Fehr et al., 1995; Das et al., 1996; Hirayama et al., 1997; Ma et al., 2004; Su et al., 2011; Wen et al., 2012; Lin et al., 2014). It is known that HA can produce singlet oxygen and semi-quinone free radicals under light irradiation (Zhou et al., 2005). However, studies on the photodynamic antifungal activity of HA were limited. HA was reported to exhibit promising activity against *C. albicans* (Ma et al., 2004), whereas the detailed mechanism of this photodynamic antifungal agent against *C. albicans* and the *in vivo* evidence of its efficacy remain to be delineated.

To investigate the antifungal mechanism and the antimicrobial activity of HA in a murine model of cutaneous *C. albicans* infections, the following determinations were performed. First, HA-mediated photodynamic inactivation of *C. albicans* was investigated. To elucidate the mechanism involved in the phototoxicity of HA and the form of cell death, ROS production, plasma membrane potential and integrity, and a variety of apoptosis hallmarks, including mitochondrial transmembrane potential, metacaspase activity, DNA fragmentation, nuclear condensation, and cytosolic and mitochondrial calcium, were determined. What’s more, the *in vivo* anti – *C. albicans* activity of HA-mediated aPDT was also investigated in a mouse model of skin wound infection.

## Materials and Methods

### Strains, Cultures, and Chemicals

Three *C. albicans* strains (SC5314, ATCC18804 and 07318) were used in this study. All these strains were routinely grown overnight at 30°C in YPD medium that consists of 1% yeast extract (Oxoid, Basingstoke, England), 2% peptone (Solarbio, Beijing, China) and 2% dextrose (Solarbio). Hypocrellin A (HA) was purchased from Tauto Biotech (Shanghai, China). The structure and UV-Vis absorption spectrum of HA were shown in Supplementary Figure S12. A stock solution of HA (1.0 mg/ml) was prepared in 100% dimethyl sulfoxide (DMSO). The solution was filter-sterilized and stored at −20∘C in the dark for not more than 1 week before use.

### Antifungal Activity Assay

Overnight cultures were refreshed in YPD medium at an OD_600_ of 0.1, then cells were grown to log phase and treated with 0.5 and 1.0 μg/ml HA for 30 min under a 8 W incandescent lamp (wavelength range: 400–780 nm) in an orbital shaker (HZQ-X100, Peiying Instrument Co., Ltd., Suzhou, Taicang, China). The distance between *C. albicans* cultures and the light source was approximately 20 cm, and the light intensity at the treatment site was 1128 lux. After that, the cultures continued to be incubated for 3 h in the dark. Next, cells were harvested, and approximately 10^3^ *C. albicans* cells were spread onto YPD agar plates. Colony-forming units (CFUs) were counted after aerobic incubation for 24 h at 30∘C. The percentage survival was measured relative to the untreated cells. This experiment was independently repeated three times.

### Intracellular ROS Measurement

The ROS levels in *C. albicans* cells were analyzed using 2′,7′- dichlorofluorescein diacetate (DCFH-DA). The loaded DCFH-DA can be converted into the membrane-impermeable agent DCFH by intracellular esterase, which can then be rapidly oxidized by ROS into its fluorescent derivative, DCF. The fluorescence intensities of DCF can indicate the levels of ROS production (Jia et al., 2014). In brief, the overnight *C. albicans* cultures were diluted to 10^6^ cells/ml in YPD medium. After incubation to log phase, cells were treated with HA for 30 min under light. Then the cells were incubated for the subsequent 3 h in the dark. Next, *C. albicans* cells were collected, washed once with YPD medium, and stained with 10 μM DCFH-DA for 30 min in darkness. The green fluorescence intensity of resuspended cells was analyzed using a FACSCalibur flow cytometer (Becton Dickinson, United States).

### Plasma Membrane Potential Evaluation

The plasma membrane potential changes were assessed using the membrane potential molecular probe, DiBAC_4_(3) (bis-(1,3-dibarbituric acid)-trimethine oxanol), as described before (Li et al., 2016) with some modification. Briefly, overnight cultures at an OD_600_ of 0.1 were grown to mid-exponential phase, then treated by HA according to the above mentioned procedures. After that, the treated *C. albicans* was harvested by centrifugation and washed thrice with PBS. The membrane potential sensitive dye DiBAC_4_(3) was added at a final concentration of 20 μg/ml in PBS, and the samples were incubated for 30 min at 37°C in the dark. The percentage of depolarized fluorescent *C. albicans* cells in the suspension was determined by flow cytometry.

### Cell Membrane Integrity Analysis

Cell membrane integrity analysis was performed according to Li et al. (2016) with a few modifications. Briefly, the treated *C. albicans* cells were harvested, and incubated with propidium iodide (PI) at a final concentration of 10 μg/ml for 30 min at 4°C in the dark. The *C. albicans* cell membrane integrity after HA treatment was determined by flow cytometry.

### Mitochondrial Membrane Potential Assays

5,5′,6,6′-tetrachloro-1,1′,3,3′-tetraethyl-benzimidazolyl carbocy- anine iodide (JC-1) was used to evaluate the changes of mitochondrial membrane potential (Jia et al., 2019). Specifically, the HA-treated *C. albicans* cells were harvested, washed, and stained with 2.5 μg/ml JC-1 for 20 min in the dark. After washed twice in PBS, the cells were analyzed by flow cytometry. The ratio of the fluorescence intensities of aggregates JC-1 (FL2) to monomer (FL1) was calculated.

### Metacaspase Activation Assay

Metacaspase activation was detected using the CaspACE FITC-VAD-FMK *in situ* marker (Promega) (Tian et al., 2017). The treated *C. albicans* cells were collected and washed. Next, the cells were stained with 5 μM CaspACE FITC-VAD-FMK at 37∘C for 20 min in darkness. After that, the cells were washed again and analyzed using flow cytometry.

### Determination of DNA Fragmentation

DNA fragmentation was examined using TUNEL staining (Jia et al., 2019). The treated cells were collected and washed with PBS, then fixed in 3.6% paraformaldehyde for 30 min, and permeabilized on ice for 2 min. After that, the cells were washed again and stained using an *in situ* cell death detection kit for 1 h at 37∘C, Next, the cells were assessed by flow cytometry.

### Nuclear Condensation and Cell Death Analyses

Nuclear condensation, and the apoptotic or necrotic cells were evaluated by Apoptosis and Necrosis Assay kit (Beyotime, China) (Song et al., 2011). In brief, the treated cells were harvested, washed, and stained with 5 μg/ml Hoechst33342 and 5 μg/ml propidium iodide (PI) for 20–30 min at 4°C. After that, the cells were collected, washed once, and examined using a Nikon Intensilight C-HGFI microscope (Nikon, Japan).

### Cytosolic and Mitochondrial Calcium Determination

The changes of cytosolic and mitochondrial calcium levels were respectively detected using Fluo-3 AM and Rhod-2 AM (Tian et al., 2017; Jia et al., 2019). Briefly, the treated *C. albicans* cells were collected, washed twice, and then resuspended in 500 μl HBSS buffer. For measuring cytosolic calcium contents, Fluo-3 AM was added to a final concentration of 2 μM. After incubation at 30°C for 40 min in darkness, cells were washed once, resuspended in 600 μl HBSS and incubated at 30°C for further 20 min. For mitochondrial calcium determination, Rhod-2 AM was added at a final concentration of 4 μM, and incubated at 37°C for 30 min in the dark. The fluorescence intensities of Fluo-3 AM and Rhod-2 AM were immediately examined using flow cytometry.

### Mouse Model of Skin Wound Infection

**FIGURE 7:**
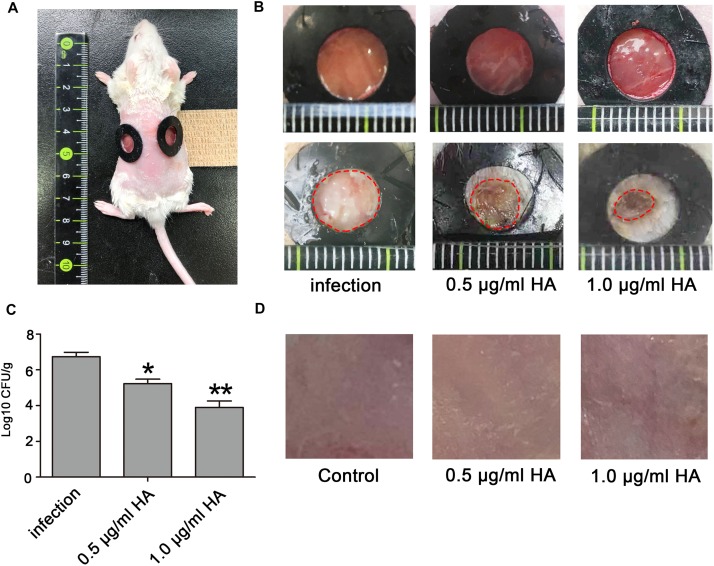
The photodynamic antifungal efficacy of HA in treating cutaneous *C. albicans* infections. **(A)** The 0.8 cm diameter wounds were created by removal of the full-thickness skin on the shaved back of the mice. **(B)** *C. albicans* skin infections and damage were observed on day 8. The mice in this experiment were divided into three groups: *C. albicans* infection group (*n* = 6), *C. albicans* infection + 0.5 μg/ml HA group (*n* = 6), and *C. albicans* infection + 1.0 μg/ml HA group (*n* = 6). In the *C. albicans* infection group, the mice were just subjected to *C. albicans* infection without HA addition. In the *C. albicans* infection + HA groups, 0.5 or 1.0 μg/ml HA and illumination for 30 min were performed at 30 min post-infection. The treatments in each group were repeated in the following 6 days. On day 8, *C. albicans* skin infections were evaluated. **(C)** The fungal burden was evaluated in the infected skin wounds on day 8. The mouse wounds were surgically excised and homogenized in PBS to release *C. albicans*. After that, the suspensions were subjected to being streaked on YPD agar plates to count CFUs after 24–48 h of incubation at 30∘C. **(D)** Toxicity of HA was observed in normal mouse skin. 0.5 or 1.0 μg/ml HA and illumination for 30 min daily were conducted on the shaved back of the mice for consecutive 7 days. ^*^P < 0.05 and ^∗∗^*P* < 0.01.

Seven to eight-week-old ICR female mice (25–30 g) were used to assess the efficacy of HA in treating *C. albcians* skin infection. Before the creation of wounds, the mice were anesthetized by intraperitoneal injection of pentobarbital sodium (50 mg/kg) and then shaved on the dorsal surface. Mouse skin was removed by scissors to create wounds. Each wound measured approximately 0.8 cm by 0.8 cm (Figure 7A). With a pipette tip, the surface of each wound was inoculated with one drop (25 μl) of the prepared culture suspension containing 10^6^ CFU of *C. albicans*, which was smeared onto the wound surface with an inoculating loop. After that, the mice were placed into three groups: *C. albicans* infection group, *C. albicans* infection + 0.5 μg/ml HA group, and *C. albicans* infection + 1.0 μg/ml HA group. In HA treatment groups, the wounds were supplemented with the indicated concentrations of HA under light illumination for 30 min after 30 min infection. *C. albicans* infection and HA treatment were repeated on the following 6 days. Six mice were used for each group. The observation of *C. albicans* infection on the mice wounds was conducted on day 8. To measure the fungal burden, the mouse wounds were surgically excised, weighed, and homogenized in PBS. Next, the suspensions containing tissues and *C. albicans* were subjected to serial 10-fold dilutions and then spread onto YPD agar plates supplemented with streptomycin and ampicillin for CFUs counting after 24–48 h of incubation at 30∘C. To evaluate the toxicity of HA, mouse skins were smeared with HA at concentrations of 0.5 and 1.0 μg/ml with 30 min illumination once a day for 7 days. Six mice were included in each group. The skins were observed on day 8.

### Statistical Analysis

Every experiment was independently performed at least three times, and the data were expressed as mean ± SD. The results were analyzed by SPSS software, using Duncan’s multiple range test following one-way ANOVA or independent sample *t*-test. Asterisks were used to indicate the statistically significance (^*^P < 0.05, ^∗∗^*P* < 0.01 and ^∗∗∗^*P* < 0.001).

## Results

### HA-Mediated aPDT Decreases *C. albicans* Survival

**FIGURE 1:**
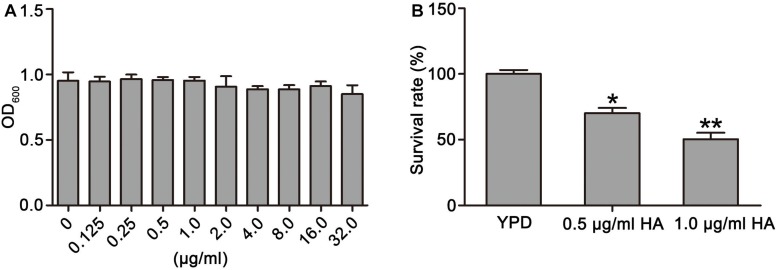
Effects of HA on growth and survival of *C. albicans* were observed. **(A)** The OD_600_ value was measured after treatment by different concentrations of HA without illumination for 24 h. **(B)** Survival rate was determined after HA treatment with illumination for 30 min by counting colony-forming units (CFUs). Data were shown as mean ± SD (*n* = 5). ^*^P < 0.05 and ^∗∗^*P* < 0.01.

To observe the effect of HA on *C. albicans* survival, SC5314 strain was treated with HA with or without light irradiation. Results showed that HA treatment without illumination did not cause significant cytotoxicity in the *C. albicans* cells at the tested concentration range (Figure 1A). However, when illumination was employed for 30 min, the survival rate of the HA-treated *C. albicans* cells respectively decreased to 70.19 ± 4.87 and 51.24 ± 4.39% compared with the control group (100 ± 3%; *P* < 0.05 and *P* < 0.01; Figure 1B), indicating that HA-mediated aPDT affected *C. albicans* survival. Moreover, the efficacy of HA against *C. albicans* was better than fluconazole (Supplementary Figure S13). To further substantiate the antifungal activity of HA against *C. albicans*, the effect of HA-mediated aPDT on two clinical *C. albicans* strains, ATCC18804 and 07318, was observed. In line with the above results, 1.0 μg/ml HA inhibited the survival of these two strains (67.31 ± 7.02 vs. 97.67 ± 6.8%, *P* < 0.05 for ATCC18804 strain; 65.4 ± 5.3 vs. 100 ± 10%, and *P* < 0.05 for 07318 strain; Supplementary Figure S1).

### HA Treatment Increases ROS Levels in *C. albicans*

**FIGURE 2:**
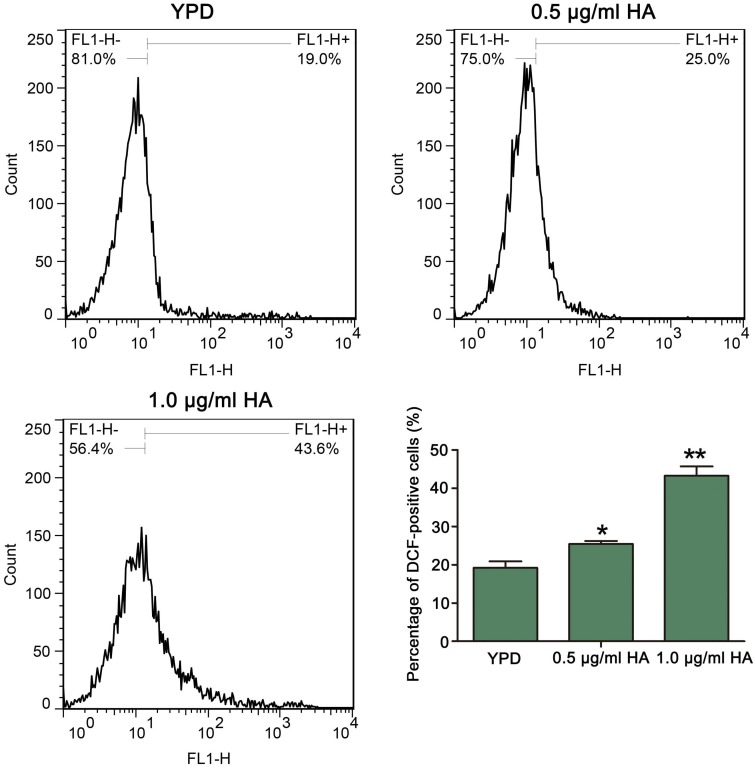
Reactive oxygen species levels were analyzed by flow cytometry using DCFH-DA after treatment by HA plus illumination for 30 min. The histogram exhibited the percentage of DCF-positive cells, and data were presented as mean ± SD (*n* = 3). ^*^P < 0.05 and ^∗∗^*P* < 0.01.

Previous study demonstrated that HA-mediated aPDT can induce ROS production in *C. albicans* cells (Chang et al., 2015). To confirm it, the ROS levels after treatment by HA were analyzed. As expected, compared with the control (19.2 ± 1.71%), HA treatment significantly elevated the percentage of DCF-positive cells in a dose-dependent manner (25.42 ± 0.79 and 43.25 ± 2.44%, respectively; *P* < 0.05 and *P* < 0.01; Figure 2). To further verify it, the effect of HA on ROS level was also observed in the above mentioned two clinical strains. As shown in Supplementary Figure S2, HA treatment led to a significant increase in the percentage of DCF-positive *C. albicans* cells compared with that in the control (35.97 ± 1.8 vs. 2.65 ± 1.32%, and *P* < 0.05 for ATCC18804 strain; 13.62 ± 0.56 vs. 6.41 ± 0.51%, and *P* < 0.05 for 07318 strain; Supplementary Figure S2). Taken together, HA addition led to ROS accumulation in *C. albicans* cells.

### HA Treatment Disrupts Plasma Membrane in *C. albicans*

**FIGURE 3:**
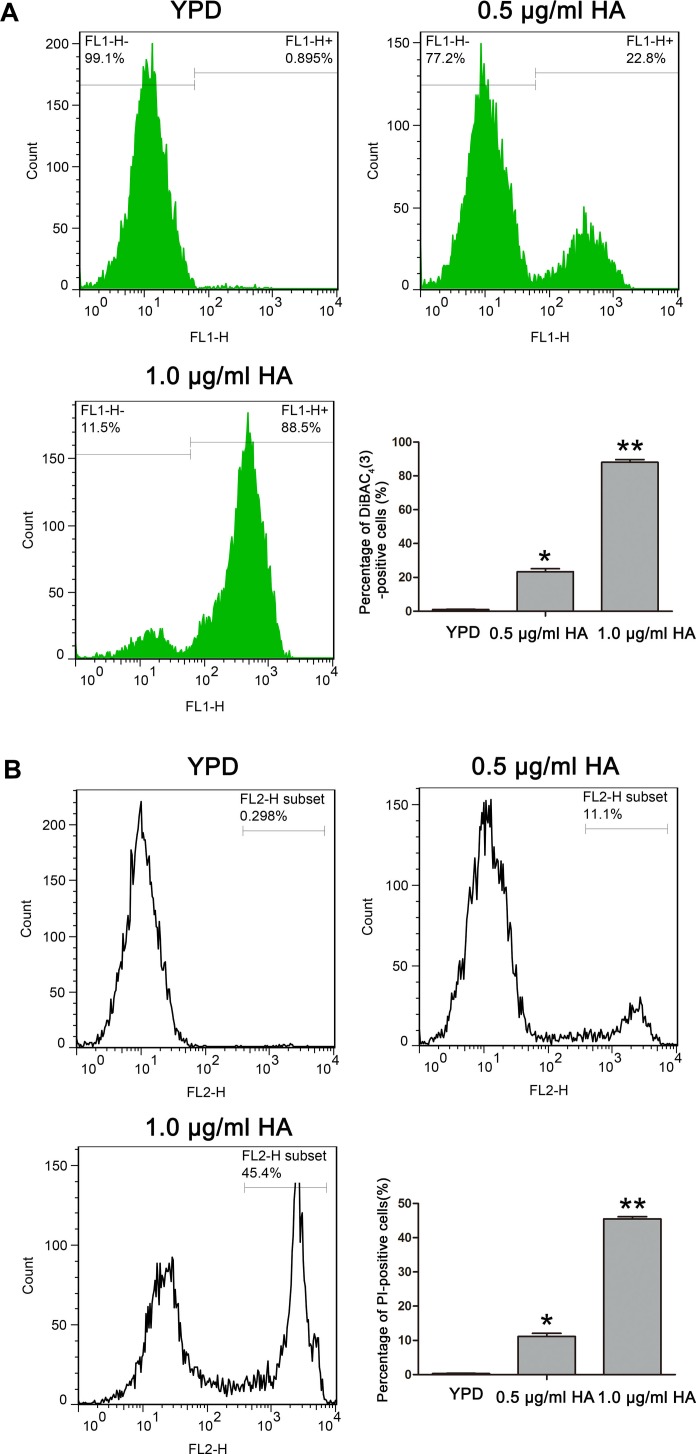
Effect of HA treatment on cell membrane in *C. albicans* cells. **(A)** The changes of cell membrane potential were assessed in HA-treated *C. albicans* cells using DiBAC_4_ (3) staining. The histogram was the quantitative analysis of DiBAC_4_ (3)-positive cells, and data were exhibited as mean ± SD (*n* = 3). ^*^P < 0.05 and ^∗∗^*P* < 0.01. **(B)** Cell membrane integrity was evaluated using PI staining. The percentage of PI-positive cells was displayed in the histogram, and data were shown mean ± SD (*n* = 3). ^*^P < 0.05 and ^∗∗^*P* < 0.01.

Many studies demonstrated that HA-induced ROS can react with lots of biomolecules, including lipids, proteins and so on, to mediate photodynamic inactivation of tumor cells (Zhang et al., 2018; Wang et al., 2019), and cell membrane was one of the main targets (Wang et al., 2019). To examine the role of HA in *C. albicans* cell membrane, the cell membrane potential and integrity in SC5314 strain were respectively evaluated using DiBAC_4_(3) and PI staining. DiBAC_4_ (3) is a plasma membrane potential-sensitive fluorescent probe. In the normal cells, it is confined to the outer regions of the plasma membrane. However, cell membrane depolarization can result in its influx, where the probe binds to lipid-rich compounds and leads to an increasing fluorescence (Nuding et al., 2006). As shown in Figure 3A, the percentage of cells that were strained by DiBAC_4_ (3) was significantly increased after HA treatment (24.08 ± 2.07 and 88.9 ± 0.52%, respectively) compared with the control (0.97 ± 0.18%; *P* < 0.05 and *P* < 0.01, respectively), indicating that HA-mediated aPDT caused a remarkable decrease in *C. albicans* plasma membrane potential. PI is a DNA-intercalating fluorescent dye that fails to enter the intact cells. However, when the cell membrane is damaged, PI probe could enter into the cytoplasm and bind to DNA. This fluorescent probe can be utilized to study the cell membrane integrity. In this study, PI staining analysis showed that there was a significant increase in the fluorescence signals after HA treatment (11.17 ± 0.9 and 45.5 ± 0.65%, respectively) compared with the control (0.33 ± 0.07%; Figure 3B), suggesting that HA-mediated aPDT triggered cell membrane damage. To further confirm the results, the cell membrane in ATCC18804 and 07318 strains were also observed after treatment with HA. Consistent with SC5314, HA addition caused cell membrane depolarization and permeability in the two strains (Supplementary Figures S3, S4). Together, these results revealed that HA treatment affected cell membrane potential and integrity in *C. albicans* cells.

### HA Decreases Mitochondrial Membrane Potential and Triggers Metacaspase Activation

**FIGURE 4:**
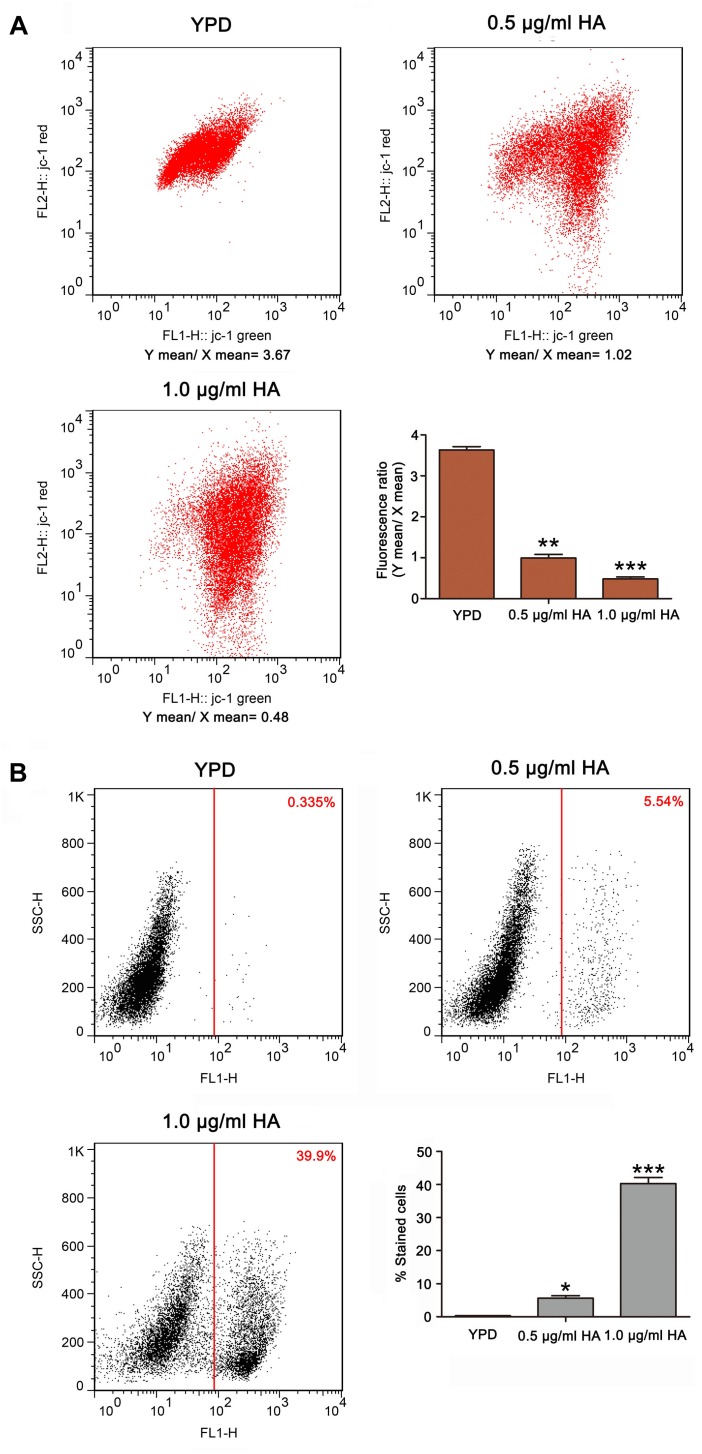
Mitochondrial membrane potential and metacaspase activation were analyzed in HA-treated *C. albicans* cells. **(A)** The mitochondrial transmembrane potential was evaluated using JC-1 staining. The histogram was the quantitative data of fluorescence ratio (*Y* mean/*X* mean). ^∗∗^*P* < 0.01 and ^∗∗∗^*P* < 0.001. **(B)** Metacaspase activity was determined using CaspACE FITC-VAD-FMK *in situ* marker. The percentage of stained cells was presented in the histogram, and data were shown as mean ± SD (*n* = 3) of three independent experiments. ^*^P < 0.05 and ^∗∗∗^*P* < 0.001.

As is known, the mitochondria are not only one of the main ROS producers, but also one target of ROS. An abnormal increase in ROS level would result in mitochondrial dysfunction (Chen et al., 2008). To evaluate mitochondrial function, the mitochondrial transmembrane potential was assessed. As anticipated, the ratio of FL2/FL1 was lower in *C. albicans* cells treated with both 0.5 μg/ml HA (0.99 ± 0.08) and 1.0 μg/ml HA (0.48 ± 0.05) than in untreated cells (3.63 ± 0.08) (Figure 4A). Previous study demonstrated that mitochondrial depolarization is associated with the opening of mitochondrial permeability transition (MPT) pores caused by HA-induced ROS generation in tumor cells. PT pore opening-mediated outer membrane rupture could lead to the release of apoptosis-related proteins to the cytosol and provoke the caspase cascade to induce apoptosis. Furthermore, HA is reported to exert an anticancer effect via inducing apoptotic cell death (Qi et al., 2019). To demonstrate the effect of HA-mediated aPDT on *C. albicans* apoptosis, the metacaspase activity was determined. Results showed that the increasing presence of metacaspase activity in *C. albicans* cells was observed from 5.65 ± 0.31 to 40.3 ± 1.83% as the concentration of HA increased in contrast to 0.315 ± 0.05% of the control cells (Figure 4B). Moreover, the mitochondrial membrane potential depolarization and metacaspase activation were also induced by HA in the two clinical strains (Supplementary Figures S5, S6), preliminarily implying that HA-mediated aPDT induced *C. albicans* apoptosis.

### HA Induces DNA Fragmentation and Nuclear Condensation in *C. albicans*

**FIGURE 5:**
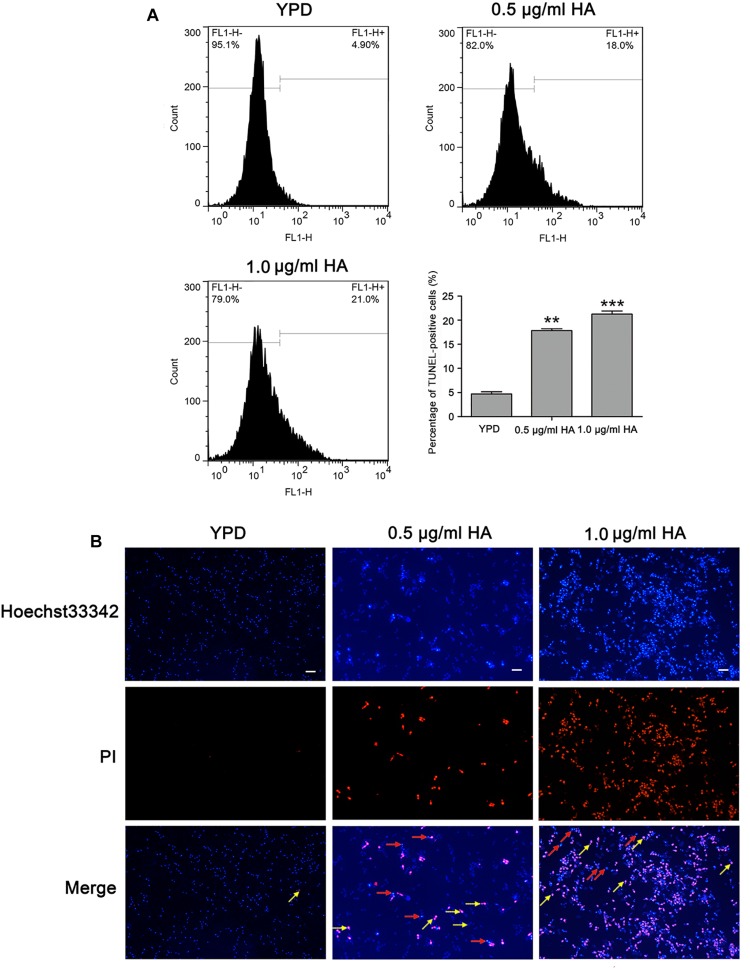
DNA fragmentation and nuclear condensation were examined in *C. albicans* cells after treatment with HA. **(A)** DNA fragmentation was analyzed by flow cytometry using TUNEL staining. The histogram showed the percentage of TUNEL-positive cells, and the values were expressed as mean ± SD (*n* = 3). ^∗∗^*P* < 0.01 and ^∗∗∗^*P* < 0.001. **(B)** Nuclear condensation was visualized with a fluorescence microscope using Hoechst33342/PI co-staining. The red arrows pointed to the apoptotic cells, and the yellow arrows indicated the necrotic cells. Scale bars = 10 μm.

To further substantiate whether HA-mediated aPDT could induce *C. albicans* cell apoptosis, two important apoptosis hallmarks, DNA fragmentation and nuclear condensation, were observed (Jia et al., 2019). Compared with the control (4.7 ± 0.44%), the percentage of TUNEL-positive cells was remarkably increased (17.83 ± 0.38 and 21.27 ± 0.64%, respectively; *P* < 0.01 and *P* < 0.001; Figure 5A), suggesting that HA treatment caused DNA fragmentation. To study whether HA could trigger nuclear condensation, the treated cells were analyzed using Hoechst 33342/PI double staining that can also indicate cell apoptosis. The normal cells would exhibit weak red/weak blue fluorescence, the apoptotic cells would show weak red/intense blue fluorescence, and the necrotic cells would display intense red/intense blue fluorescence. The intense blue fluorescence indicates nuclear condensation. Our observation showed that the HA-treated *C. albicans* cells exhibited three kinds of fluorescence, including weak red/weak blue fluorescence, weak red/intense blue fluorescence and intense red/intense blue fluorescence, suggesting that HA treatment induced nuclear condensation, and apoptosis in addition to necrosis (Figure 5B). To further substantiate the above results, TUNEL and Hoechst 33342/PI staining were also conducted in the above mentioned two clinical strains. Similar to SC5314 strain, the HA-treated ATCC18804 and 07318 strains exhibited fragmented DNA and condensed nuclei (Supplementary Figures S7, S8), revealing that HA addition induced *C. albicans* apoptosis.

### HA Elevates Cytosolic and Mitochondrial Calcium Levels

**FIGURE 6:**
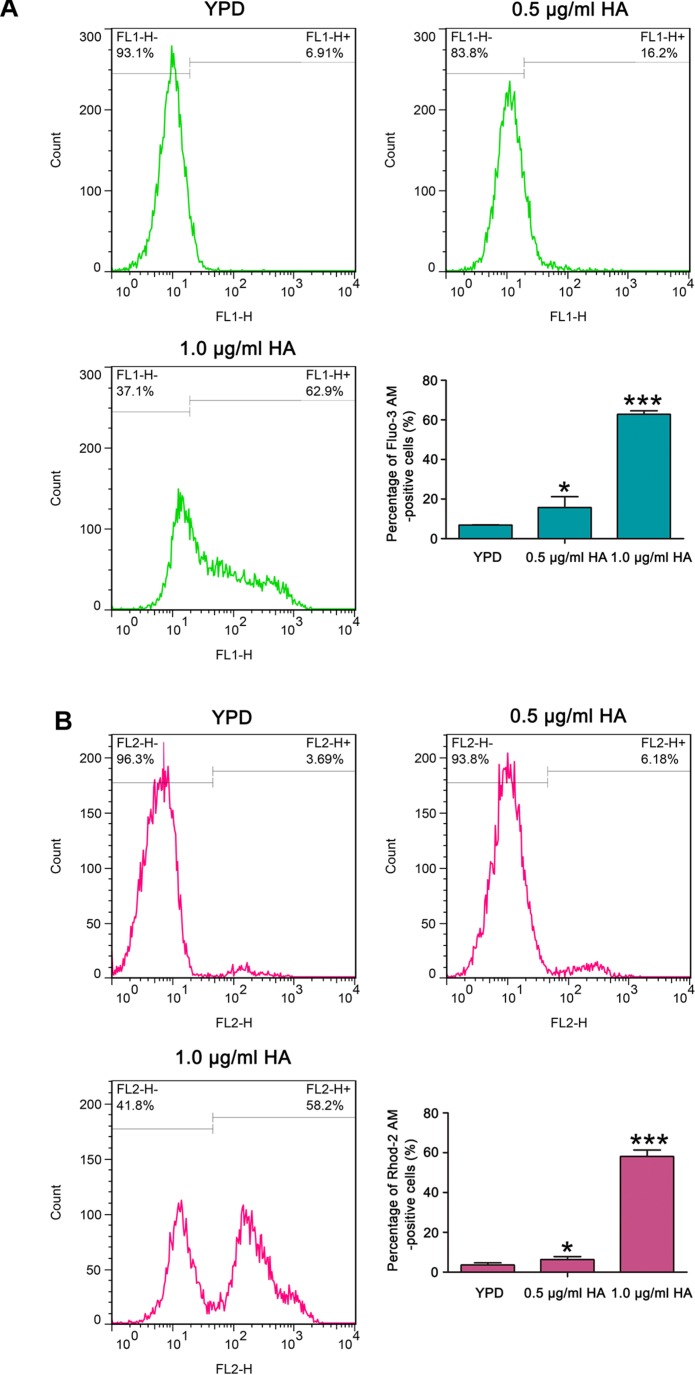
Calcium levels were assessed in the cytosol and mitochondria after HA treatment. **(A)** Cytosolic calcium contents were examined by flow cytometry via Fluo-3 AM staining. The percentage of Fluo-3 AM-positive cells were shown in the histogram, and data were shown as mean ± SD (*n* = 3). ^*^P < 0.05 and ^∗∗∗^*P* < 0.001. **(B)** Mitochondrial calcium level was evaluated using Rhod-2 AM staining. The histogram was the quantitative analysis of the percentage of Rhod-2 AM-positive cells, and the data were expressed as mean ± SD (*n* = 3). ^*^P < 0.05 and ^∗∗∗^*P* < 0.001.

It is known that calcium plays an important role in the process of apoptosis (Carraro and Bernardi, 2016). Therefore, the cytosolic and mitochondrial calcium levels were respectively determined using Fluo-3 AM and Rhod-2 AM. Data showed that the percentage of Fluo-3 AM-positive cells respectively increased to 17.9 ± 2.6 and 62.77 ± 1.8% after treatment with 0.5 and 1.0 μg/ml HA in contrast to 6.87 ± 0.08% of the control cells (Figure 6A). Moreover, compared with the control (3.58 ± 0.24%), the Rhod-2 AM-positive cells were also significantly elevated in HA-treated *C. albicans* cells (6.29 ± 0.47 and 58.13 ± 0.21%, respectively; *P* < 0.05 and *P* < 0.001; Figure 6B). Similar calcium changes were also observed in the two clinical strains after treatment with 1.0 μg/ml HA (Supplementary Figures S9, S10). All these results indicated that HA-mediated aPDT caused cytosolic and mitochondrial calcium accumulation in *C. albicans* cells.

### HA-Mediated aPDT Alleviates Cutaneous *C. albicans* Infection in Mice

To *in vivo* examine the efficacy of HA for prophylaxis of *C. albicans* infections, aPDT using HA was initiated at 30 min after fungal inoculation. *C. albicans* infection and subsequent HA treatment were conducted once per day over seven consecutive days. Results showed that HA-mediated aPDT remarkably alleviated the *C. albicans* skin infections as evidenced by reduced wound infection size (Figure 7B), and decreased *C. albicans* burden in the infected mouse skin wounds (Figure 7C). To detect the toxicity of HA, non-infected normal mouse skin was smeared with HA at concentrations of 0.5 and 1.0 μg/ml and irradiated for 30 min, which were repeated for 7 days. Observation showed that there was no significant difference between the treated and untreated mouse skins (Figure 7D), indicating that HA was not toxic to the mouse skin below a concentration of 1.0 μg/ml.

## Discussion

*Candida albicans* is a major opportunistic human pathogen that could cause both superficial and disseminated forms of infections. The increasing emergence of antifungal resistance among *Candida* spp. leads to a growing interest in the antifungal effects of aPDT as a therapy for localized candidiasis, and many PSs-dependent aPDT was demonstrated to be effective against *C. albicans.* For instance, Mang et al. (2010) reported that Photofrin-induced aPDT can significantly eliminate normal and antifungal resistant *Candida* species. Dovigo et al. (2013) demonstrated that curcumin-mediated aPDT could inactivate *C. albicans* in a murine model of oral candidiasis. Methylene blue-mediated aPDT decreased *C. albicans* growth rate, and the ability to form germ tube (Kato et al., 2013). Photodynamic antimicrobial chemotherapy using chlorin e6 significantly inhibited biofilm formation by *C. albicans* through increasing ROS production and membrane permeability (Carvalho et al., 2018). Photodynamic inactivation of *C. albicans* by a cationic porphyrin-TriP [4] was realized by damaging cytoplasmic membrane (Lambrechts et al., 2005). HA, as a type of PS, has been reported to be able to carry out aPDT of both Gram-positive and Gram-negative bacteria when combined with CaCl_2_ or MgCl_2_ (Su et al., 2011). Ma et al. documented that HA exhibits effective activity against *C. albicans* (Ma et al., 2004). However, the detailed mechanisms of HA against *C. albicans* and *in vivo* application of aPDT using HA have not yet been elucidated.

In this study, we found that HA-mediated aPDT was able to promote antifungal effects against *C. albicans*. The combination of 0.5 or 1.0 μg/ml HA with light irradiation could lead to a significant decrease in the viable *C. albicans* counts compared with the control. In contrast, HA at the tested concentrations did not have an obvious influence on the growth of *C. albicans* in the absence of light irradiation, which was in agreement with previous studies (Su et al., 2011). Numerous studies reported that interaction of light results in excitation of HA in a triplet state that is capable of directly reacting with cellular substrate to form radicals, which subsequently transfer energy to oxygen to generate ROS (Zhang et al., 1998; Lu et al., 2006). The produced ROS play an important role in the photodynamic antimicrobial process of HA (Su et al., 2011). Our results showed that ROS generation was remarkably increased after HA treatment. ROS produced by photoactivated dyes are free radicals or non-radical species derived from oxygen, including either singlet oxygen or other ROS such as superoxide and hydroxyl radicals (Baltazar et al., 2015). To verify the implication of ROS in the growth inhibition induced by HA-mediated aPDT, one of type I scavengers – mannitol, and one of type II quenchers – histidine were respectively applied. As depicted in Supplementary Figure S15, the growth of *C. albicans* incubated with HA after illumination increased from 0.07 to 0.36/0.14 in the presence of mannitol/histidine, indicating that HA-mediated growth inhibition of *C. albicans* was related to ROS production. These ROS family could react with the cytoplasmic membrane to inactivate the membrane transport system and enzymes (Hamblin and Hasan, 2004). Previous study has reported that HA exhibits a strong photodynamic effect on the cell membrane of tumor cells (Hamblin and Hasan, 2004). Our present study also found that the cell membrane potential and integrity were disrupted in *C. albicans* cells after treatment with HA plus illumination, indicating that the structures of *C. albicans* cell wall and membrane were altered by HA-mediated aPDT, which could result in the further translocation of HA into the cells. Since we did not examine other cell structures, HA-mediated aPDT might be also in association with the damage of other cell components, such as the endoplasmic reticulum, Golgi bodies and so on.

As is known, high doses of light and high concentrations of PS can lead to necrotic cell death, while low doses tends to trigger apoptotic cell death (Lennon et al., 1991; Noodt et al., 1996; Mroz et al., 2011). Previous studies reported that HA-mediated photodynamic action is associated with mitochondria-involved apoptosis (Fu and Chu, 1989; Qi et al., 2019). Our results demonstrated that HA at the tested concentrations also induced *C. albicans* cell apoptosis in addition to necrosis, as evidenced by mitochondrial membrane potential depolarization, metacaspase activation, DNA fragmentation, and nuclear condensation. In human lung adenocarcinoma A549 cells, HA is actively taken up into the cytosol and localized to mitochondria, and HA-based photodynamic action induces apoptosis through ROS-mediated mitochondrial signaling pathway (Qi et al., 2019). In light of our present study, we speculated that HA-mediated *C. albicans* apoptosis might also be via mitochondrial intrinsic apoptosis pathway since mitochondria, as double membrane bounded organelles, are one main target of ROS. Calcium level analysis showed HA-treated cells displayed an increase in calcium accumulation in both cytosol and mitochondria. Cytosolic calcium elevation might be explained by the reason that HA treatment disrupted the structures, or transport system and enzymes of plasma membrane and intracellular membranes that allow extracellular calcium influx and/or calcium release from intracellular stores (Jia et al., 2015). The increase in mitochondrial calcium level might be caused by the transfer of cytosolic calcium, which further triggered the release of proapoptotic proteins and apoptotic cell death (Carraro and Bernardi, 2016). The up-regulation of all above hallmarks indicated aPDT via HA was correlated with apoptosis induction in *C. albcians* cells.

The efficacy of aPDT against different fungal pathogens has been confirmed *in vivo* using various animal models (Baltazar et al., 2015). For example, Dai et al. evaluated NMB plus red light-mediated aPDT in a murine cutaneous *C. albicans* infection model, and found that aPDT initiated either at 30 min or at 24 h post-infection remarkably declined the *C. albicans* burden compared to the control (Dai et al., 2011). The efficacy of Photogem and curcumin-mediated aPDT were assessed in an oral candidiasis, and results showed that these two PSs could significantly reduce *C. albicans* viability (Mima et al., 2010; Dovigo et al., 2013). Methylene blue-mediated aPDT was evaluated in a mouse model of systemic infection, and results demonstrated that the survival of mice systemically infected with *C. albicans* was significantly increased after pretreatment with methylene blue (Kato et al., 2013). In this study, to *in vivo* investigate the effect of HA-mediated aPDT in a mouse model of skin wound infection, HA plus illumination was applied after fungal inoculation for 30 min. Since an infection for 30 min cannot successfully establish the infection model, we repeated the fungal inoculation and HA treatment for seven consecutive days. Due to that the growth of *C. albicans* cells was not affected by light-only treatment compared with the control (Supplementary Figure S11), we did not include the light-only treatment group. Results showed that *C. albicans* infection without HA treatment group showed obvious wound infection. However, HA treatment group exhibited a smaller infection size and skin damage. Moreover, the skin fungal burden was significantly decreased after HA treatment. However, there is an obvious discrepancy between *in vitro* and *in vivo* obtained results in *C. albicans* reduction of survival (Supplementary Figure S14). This could be explained by the following reasons: the *in vitro* results about the percentage survival were only subjected to the effect of HA-mediated aPDT. Before observing CFUs, the number of *C. albicans* cells in different groups was adjusted to the same (approximately 10^3^), which neglected the effect of HA treatment on *C. albicans* growth. However, *in vivo* CFUs were acquired relative to the same weight of mouse skin. The CFUs were affected by both the total number of *C. albicans* cells and survival rate. Previous study demonstrated that hypocrellin photosensitizer SL052-based photodynamic therapy could elevate the numbers of immune cells and activate immune response (Korbelik et al., 2009). Therefore, we speculated the *in vivo* data may be a synergistic result of both HA-mediated aPDT and immune system activation. All these results suggested HA utilization remarkably ameliorated *C. albicans* skin wound infections. Toxicity assays exhibited that HA was non-toxic to the mouse skin at the concentration of 1.0 μg/ml HA. These results indicated that HA-mediated aPDT was effective and low-toxic in treating *C. albicans* skin infection. Currently, many photosensitizers are demonstrated to be effective in inhibiting or killing *C. albicans*, including methylene blue (Kato et al., 2013), chlorin e6 (Carvalho et al., 2018), toluidine blue, malachite green (Souza et al., 2010), porphyrin (Lambrechts et al., 2005; Gonzales et al., 2013), hypericin (Rezusta et al., 2012), imidazoacridinones (Taraszkiewicz et al., 2015), and bacteriochlorins (Huang et al., 2010). Since the efficacy of these PSs on *C. albicans* was respectively investigated in different studies. To better compare their advantages and disadvantages, they could be studied together in the future.

There are some limitations in our current study. The information from the study has not provided whether *C. albicans* can develop HA resistance with increasing numbers of passages. In addition, the present study just demonstrated the efficacy of HA in the prophylaxis of cutaneous *C. albicans* infection in mice. The effectiveness of treatment is still unclear. Many fungi, including *C. albicans*, can generate melanin that can absorb light and prevent fungi from light-dependent inactivation. In this study, *C. albicans* was not incubated in melanin inducing conditions. Therefore, further study is needed to examine the effect of melanin on HA efficacy. Nevertheless, evidence from present studies demonstrates that HA-mediated aPDT is a safe and effective therapy strategy to decrease cutaneous *C. albicans* infections.

## Data Availability

All datasets generated for this study are included in the manuscript and/or the Supplementary Files.

## Ethics Statement

Healthy ICR female mice (25–30 g) were provided by Wenzhou Medical University (License No. SCXK [ZJ] 2005-0019). The procedures involving animals complied with the Guide for the Care and Use of Laboratory Animals, which were authorized by the Animal Care and Use Committee of Wenzhou Medical University (wydw 2017-0046). Efforts were made to minimize the suffering of the mice.

## Author Contributions

YY, CW, and YZ performed most of the experiments. JZ and KX participated in measuring calcium levels and TUNEL staining. QZ, HZ, and HC carried out the animal experiments. MC participated in revising the manuscript. CJ was responsible for designing the experiments and writing the manuscript.

## Conflict of Interest Statement

The authors declare that the research was conducted in the absence of any commercial or financial relationships that could be construed as a potential conflict of interest.

## Supplementary Material

FIGURE S1 Effect of 1.0 μg/ml HA on the survival of ATCC18804 and 07318 strains. Data were shown as mean ± SD. ^*^*P* < 0.05.

FIGURE S2 Effect of 1.0 μg/ml HA on ROS production of ATCC18804 and 07318 strains. The histogram showed the percentage of DCF-positive cells, and data were presented as mean ± SD. ^*^*P* < 0.05 and ^∗∗∗^*P* < 0.001.

FIGURE S3 Effect of 1.0 μg/ml HA on cell membrane potential of ATCC18804 and 07318 strains were assessed using DiBAC4 (3) staining. The histogram was the quantitative analysis of DiBAC4 (3)-positive cells, and data were exhibited as mean ± SD. ^*^*P* < 0.05 and ^∗∗^*P* < 0.01.

FIGURE S4 Effect of 1.0 μg/ml HA on cell membrane integrity of ATCC18804 and 07318 strains were assessed using PI staining. The percentage of PI-positive cells was displayed in the histogram, and data were shown mean ± SD. ^*^*P* < 0.05 and ^∗∗^*P* < 0.01.

FIGURE S5 The mitochondrial transmembrane potential was evaluated using JC-1 staining in ATCC18804 and 07318 strains. The histogram was the quantitative data of fluorescence ratio (Y mean/X mean). ^*^*P* < 0.05.

FIGURE S6 Metacaspase activity was determined using CaspACE FITC-VAD-FMK *in situ* marker in ATCC18804 and 07318 strains. The percentage of stained cells was presented in the histogram, and data were presented as mean ± SD. ^*^*P* < 0.05.

FIGURE S7 DNA fragmentation in ATCC18804 and 07318 strains was analyzed by flow cytometry using TUNEL staining. The histogram showed the percentage of TUNEL-positive cells, and the values were expressed as mean ± SD. ^*^*P* < 0.05.

FIGURE S8 Nuclear condensation in ATCC18804 and 07318 strains was visualized with a fluorescence microscope using Hoechst33342/PI co-staining. Scale bars = 5 μm.

FIGURE S9 Effect of HA on cytosolic calcium contents were examined by flow cytometry via Fluo-3 AM staining in ATCC18804 and 07318 strains. The percentage of Fluo-3 AM-positive cells were shown in the histogram, and data were shown as mean ± SD. ^∗∗^*P* < 0.01.

FIGURE S10 Mitochondrial calcium level in ATCC18804 and 07318 strains was evaluated using Rhod-2 AM staining. The histogram was the quantitative analysis of the percentage of Rhod-2 AM-positive cells, and the data were expressed as mean ± SD. ^*^*P* < 0.05.

FIGURE S11 Effect of light-only treatment on the growth of *C. albicans*. ^*^*P* < 0.05 and ^∗∗^*P* < 0.01.

FIGURE S12 The structure and UV-Vis absorption spectrum of HA.

FIGURE S13 Effect of HA and fluconazole (FLC) on C. albicans growth. ^*^*P* < 0.05, ^∗∗^*P* < 0.01, and ^∗∗∗^*P* < 0.001.

FIGURE S14 Effect of HA treatment on colony-forming units without adjusting to the same number. ^*^*P* < 0.05.

FIGURE S15 Effect of ROS scavengers and quenchers on the photodynamic inactivation of *C. albicans* by HA. ^*^*P* < 0.05 and ^∗∗^*P* < 0.01.
