# Supplementary figures and images for: Photodynamic Antifungal Activity of Hypocrellin A Against Candida albicans
Source: Front Microbiol. 2019 Aug 6;10:1810. doi: 10.3389/fmicb.2019.01810 (PMC6691099; doi:10.3389/fmicb.2019.01810)

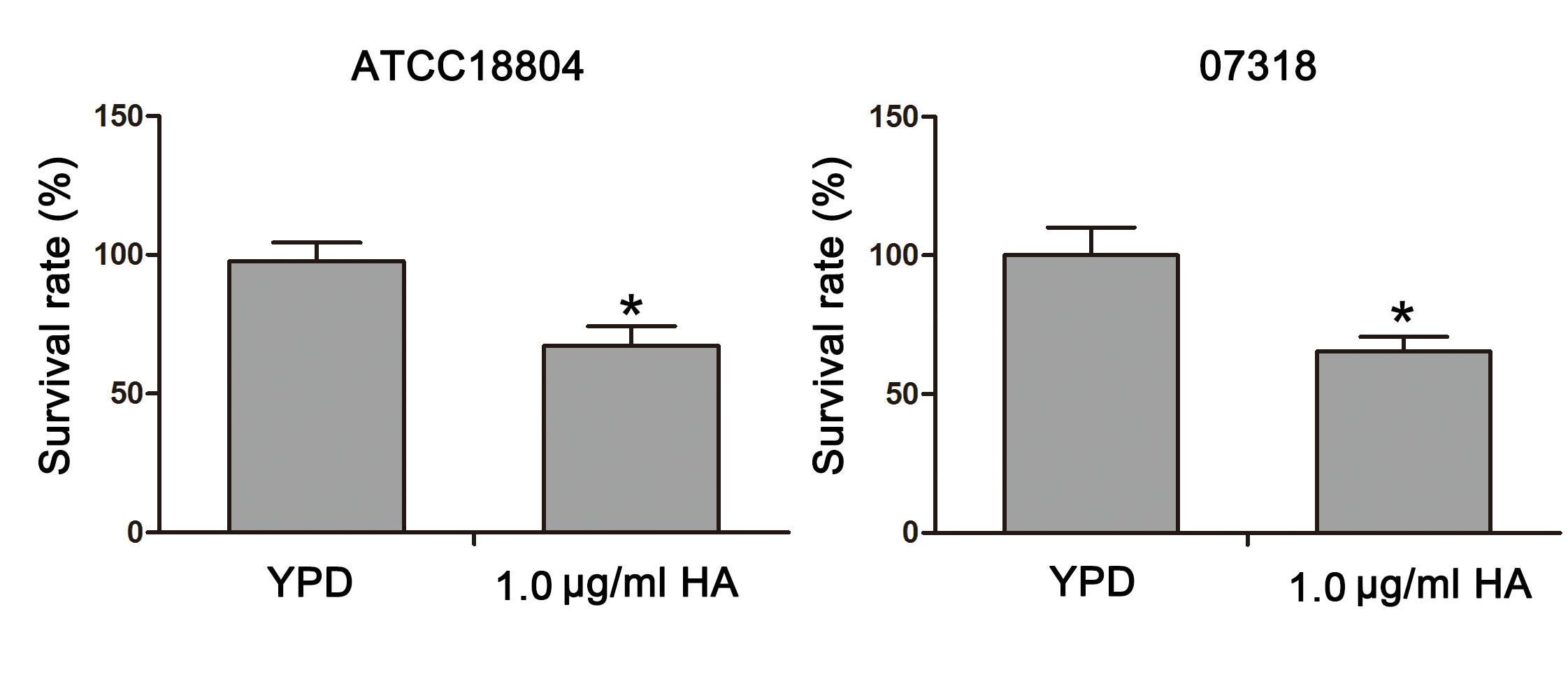

Supplement: FIGURE S1 — Effect of 1.0 μg/ml HA on the survival of ATCC18804 and 07318 strains. Data were shown as mean ± SD. *P < 0.05. [file Image_1.JPEG]

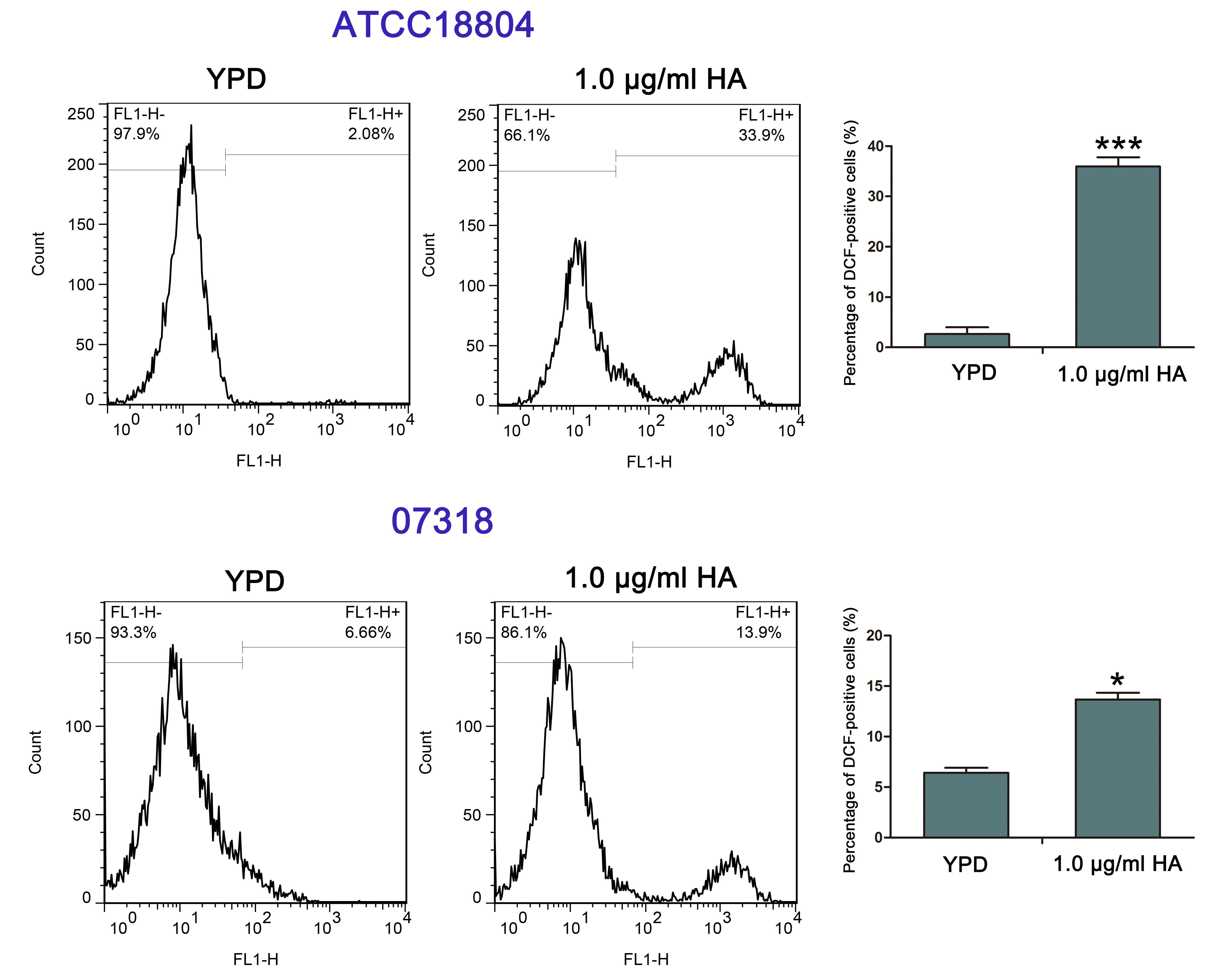

Supplement: FIGURE S2 — Effect of 1.0 μg/ml HA on ROS production of ATCC18804 and 07318 strains. The histogram showed the percentage of DCF-positive cells, and data were presented as mean ± SD. *P < 0.05 and ∗∗∗P < 0.001. [file Image_2.JPEG]

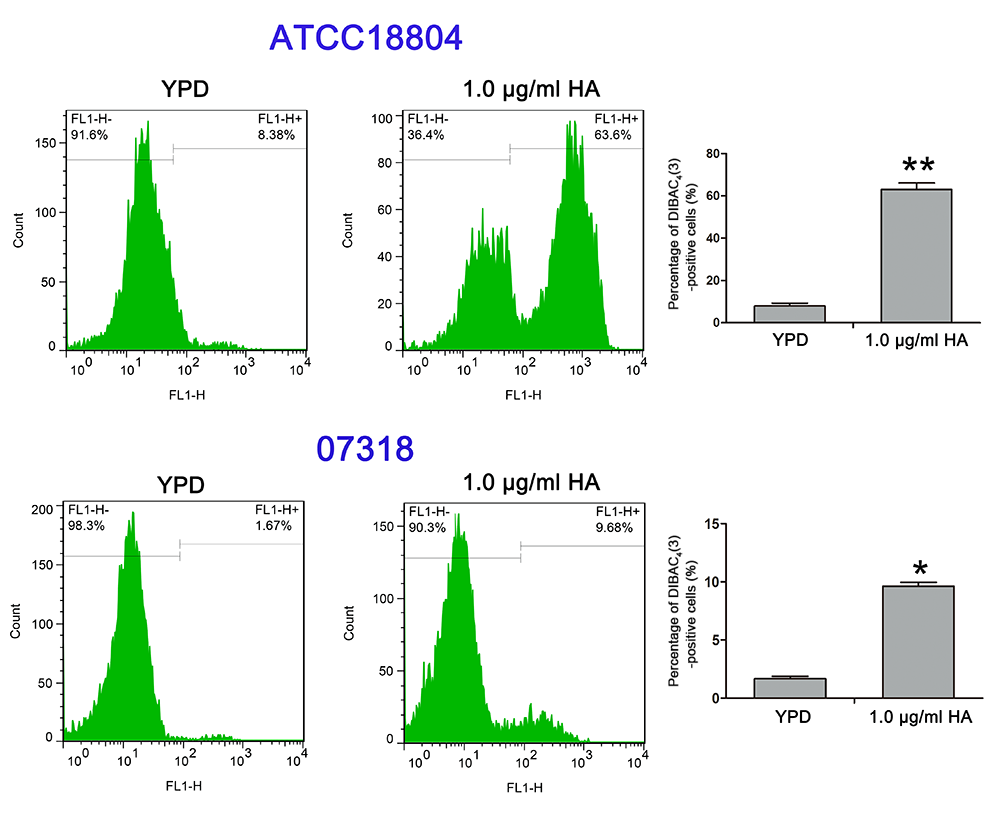

Supplement: FIGURE S3 — Effect of 1.0 μg/ml HA on cell membrane potential of ATCC18804 and 07318 strains were assessed using DiBAC4 (3) staining. The histogram was the quantitative analysis of DiBAC4 (3)-positive cells, and data were exhibited as mean ± SD. *P < 0.05 and ∗∗P < 0.01. [file Image_3.TIF]

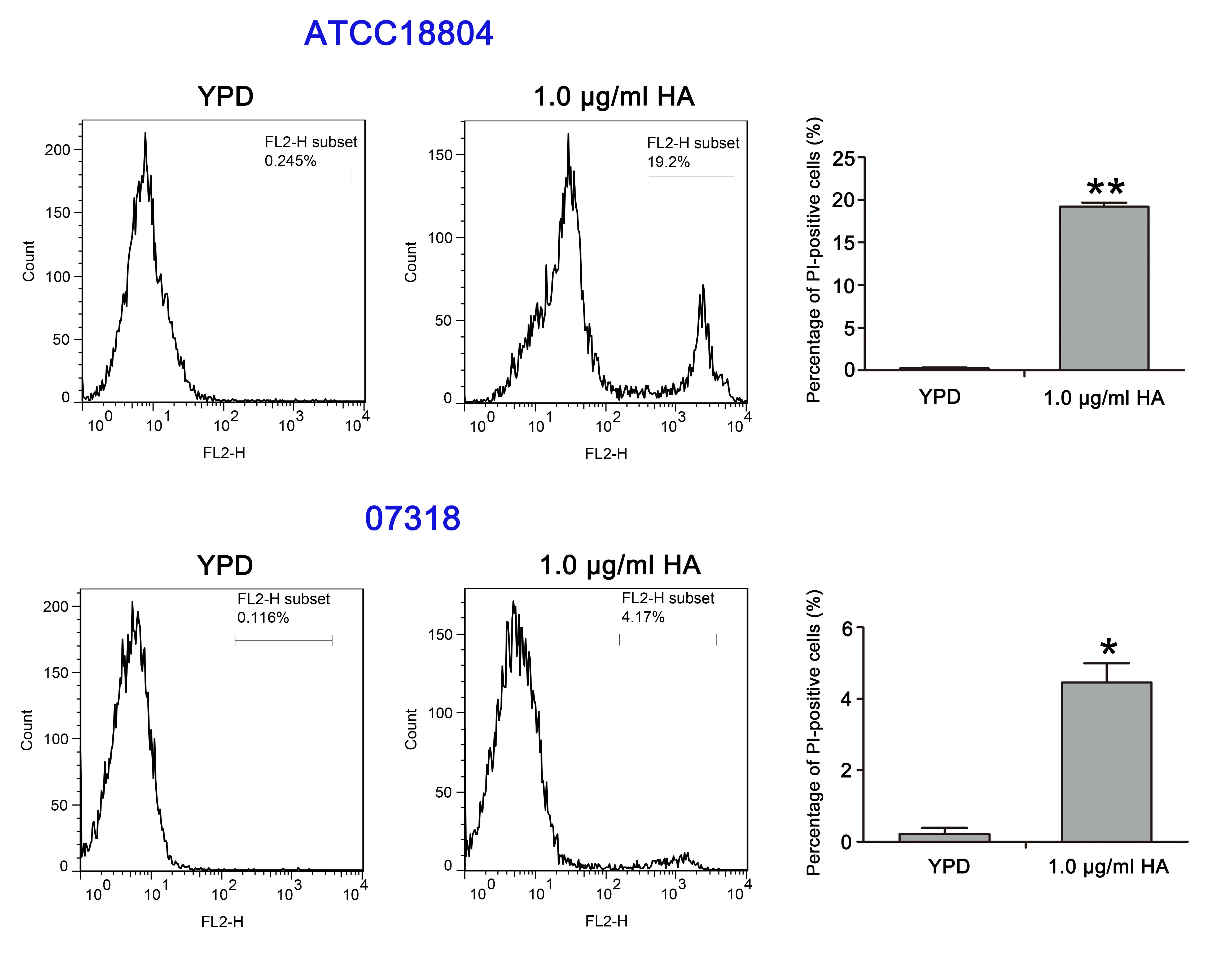

Supplement: FIGURE S4 — Effect of 1.0 μg/ml HA on cell membrane integrity of ATCC18804 and 07318 strains were assessed using PI staining. The percentage of PI-positive cells was displayed in the histogram, and data were shown mean ± SD. *P < 0.05 and ∗∗P < 0.01. [file Image_4.JPEG]

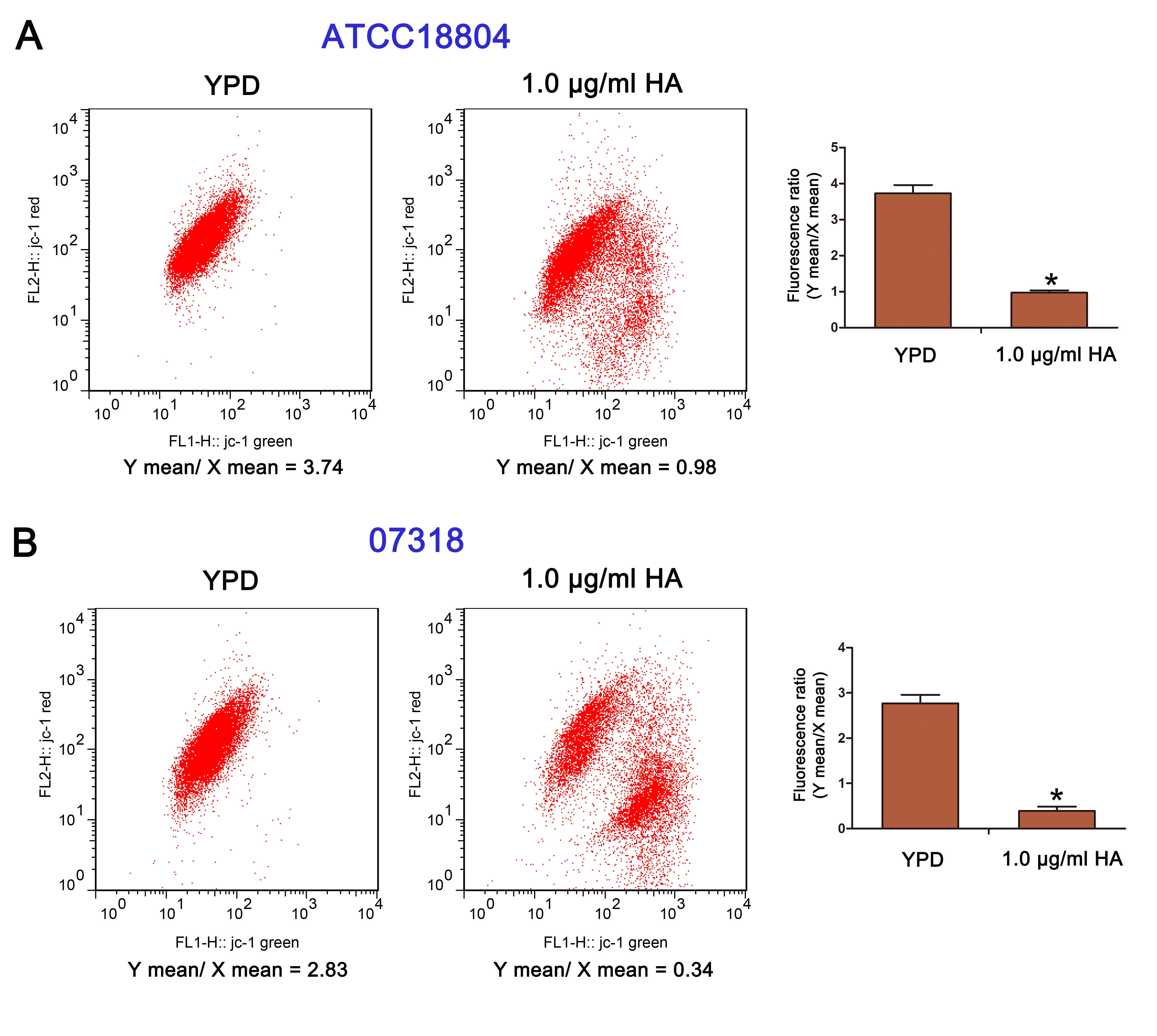

Supplement: FIGURE S5 — The mitochondrial transmembrane potential was evaluated using JC-1 staining in ATCC18804 and 07318 strains. The histogram was the quantitative data of fluorescence ratio (Y mean/X mean). *P < 0.05. [file Image_5.JPEG]

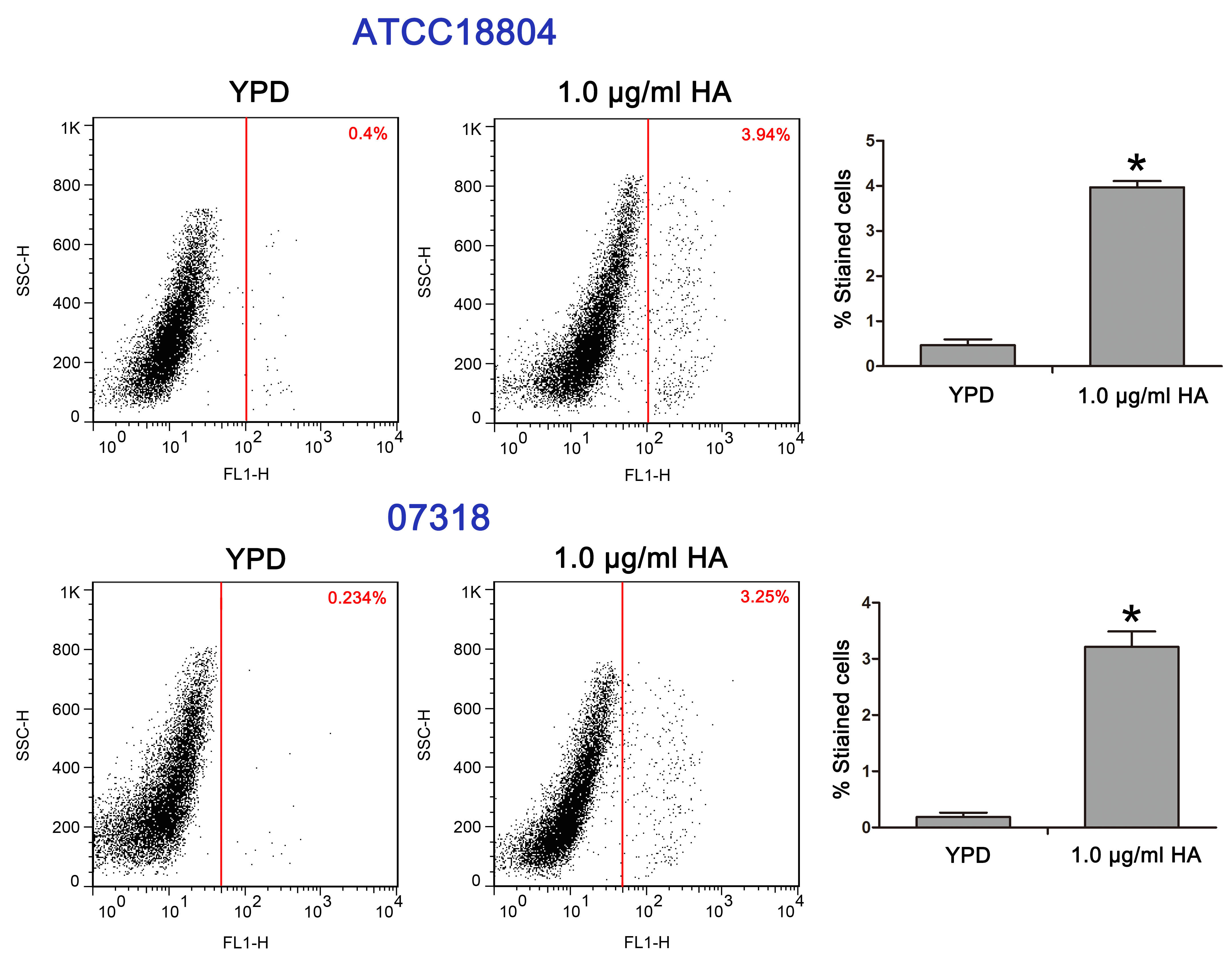

Supplement: FIGURE S6 — Metacaspase activity was determined using CaspACE FITC-VAD-FMK in situ marker in ATCC18804 and 07318 strains. The percentage of stained cells was presented in the histogram, and data were presented as mean ± SD. *P < 0.05. [file Image_6.JPEG]

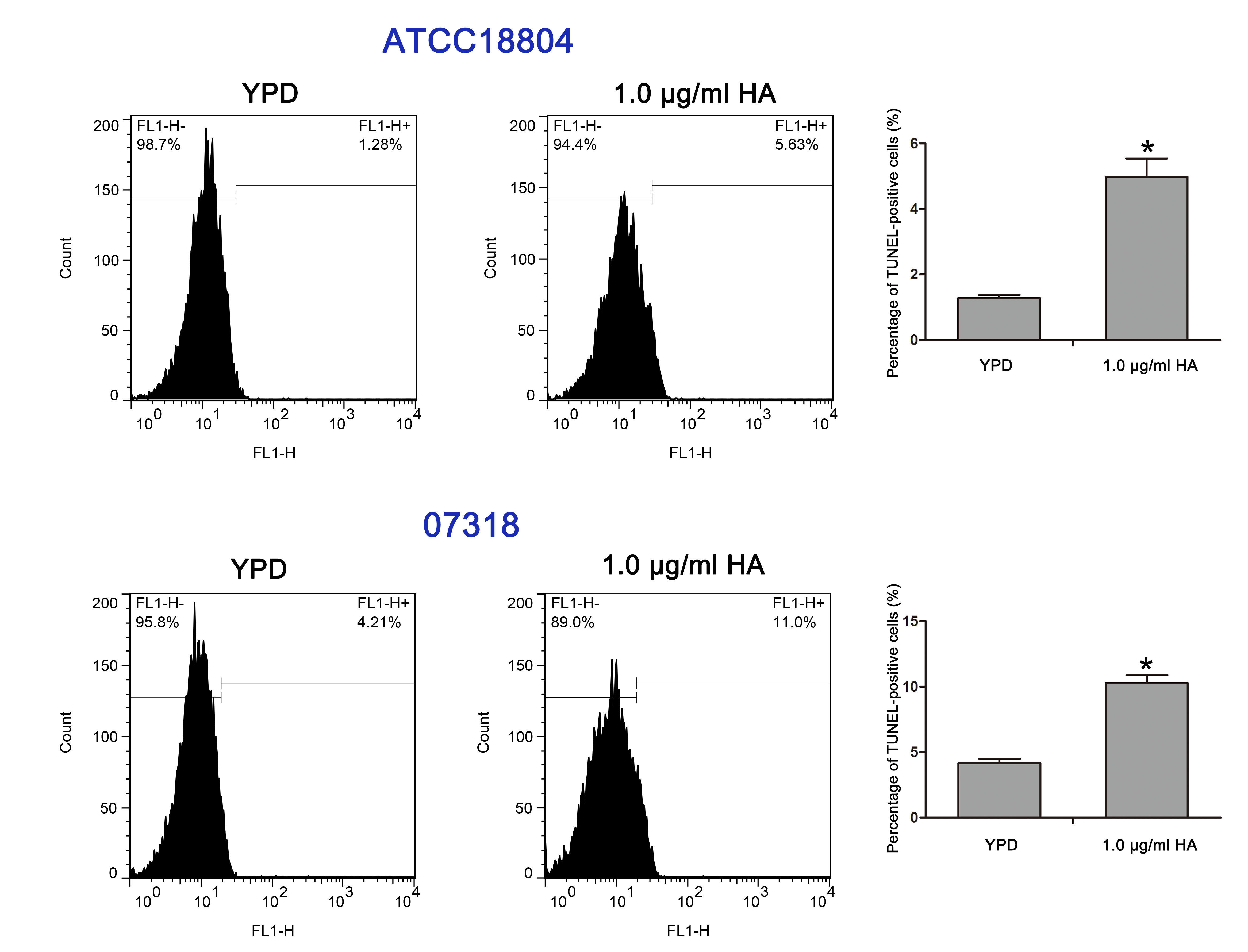

Supplement: FIGURE S7 — DNA fragmentation in ATCC18804 and 07318 strains was analyzed by flow cytometry using TUNEL staining. The histogram showed the percentage of TUNEL-positive cells, and the values were expressed as mean ± SD. *P < 0.05. [file Image_7.JPEG]

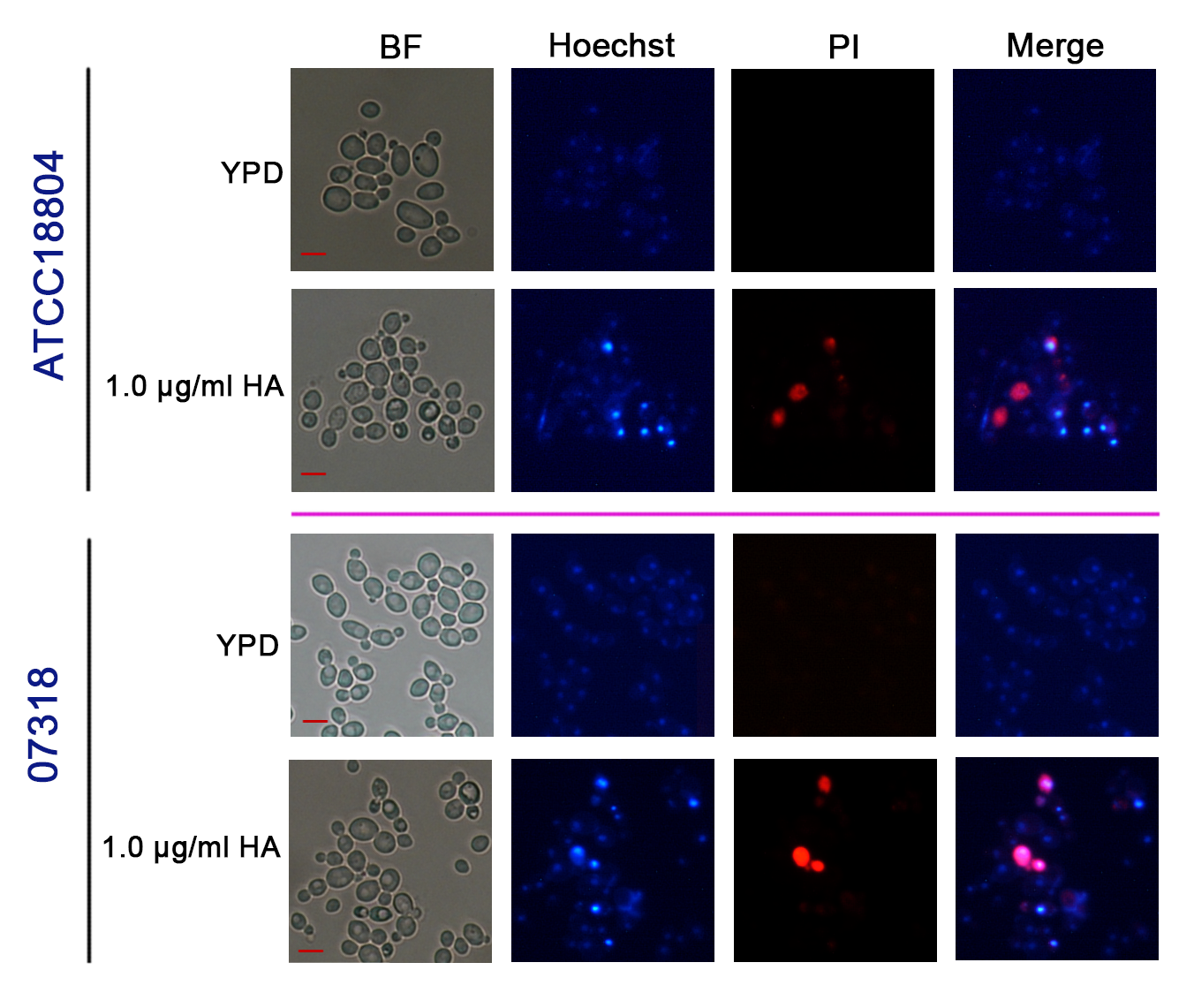

Supplement: FIGURE S8 — Nuclear condensation in ATCC18804 and 07318 strains was visualized with a fluorescence microscope using Hoechst33342/PI co-staining. Scale bars = 5 μm. [file Image_8.TIF]

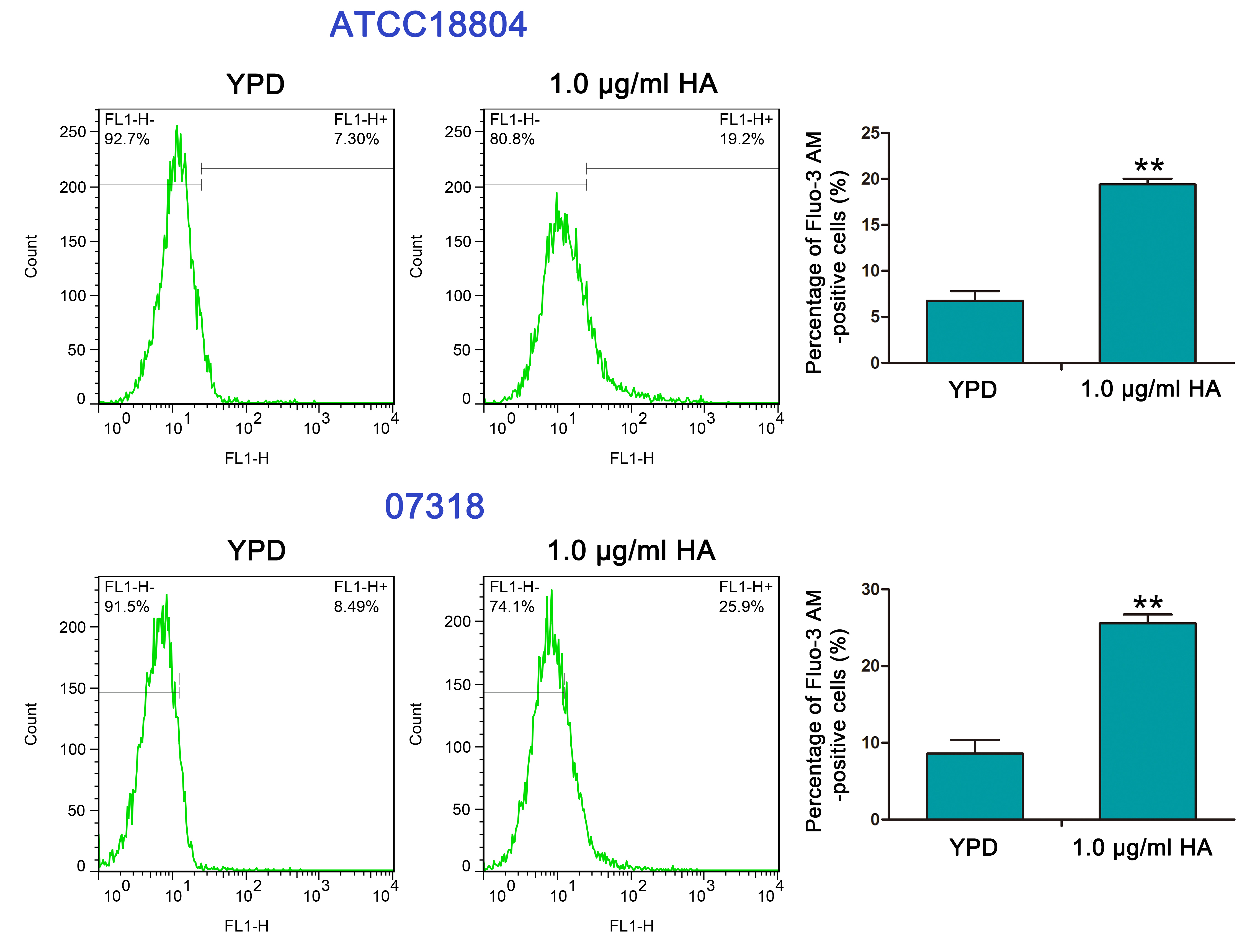

Supplement: FIGURE S9 — Effect of HA on cytosolic calcium contents were examined by flow cytometry via Fluo-3 AM staining in ATCC18804 and 07318 strains. The percentage of Fluo-3 AM-positive cells were shown in the histogram, and data were shown as mean ± SD. ∗∗P < 0.01. [file Image_9.JPEG]

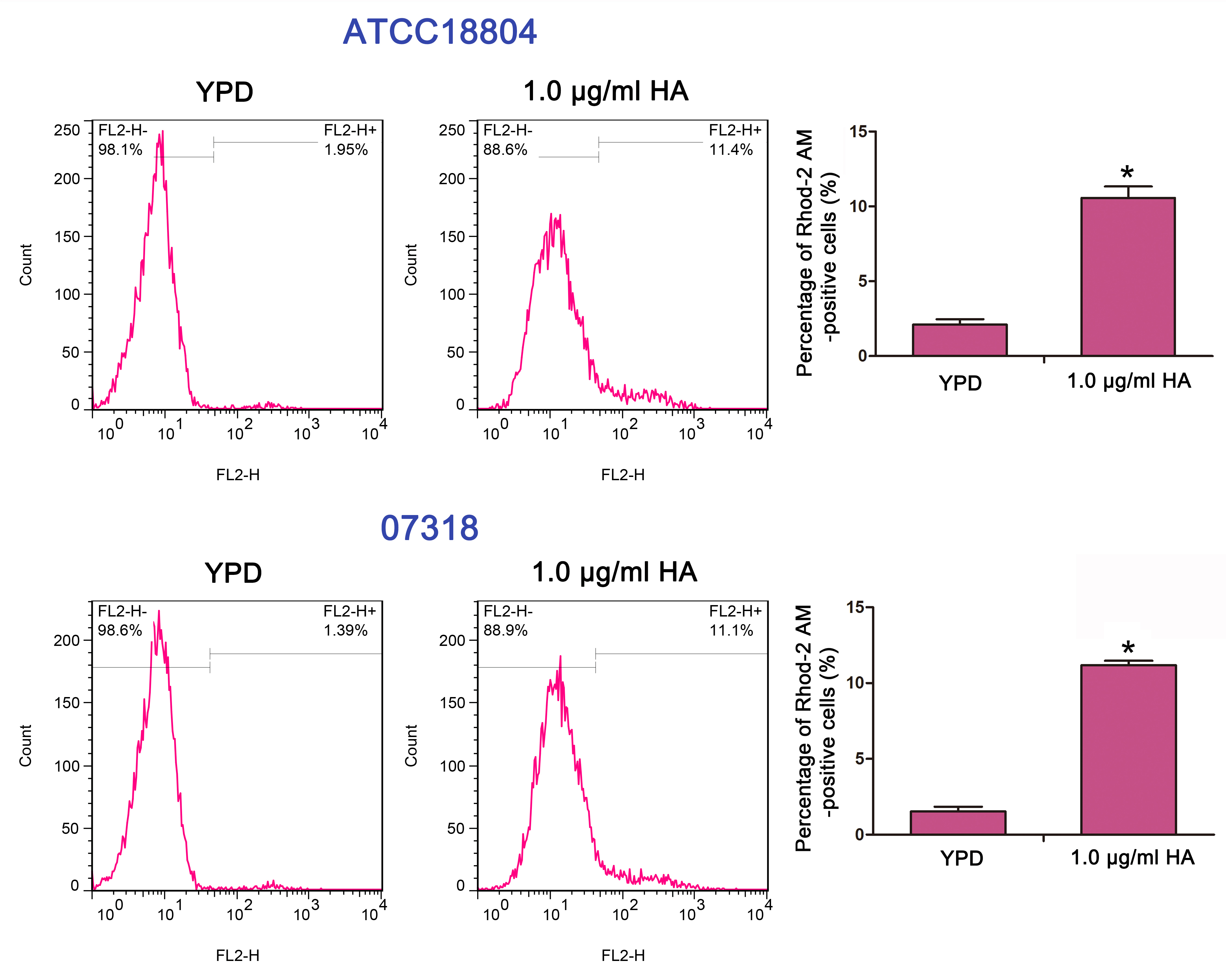

Supplement: FIGURE S10 — Mitochondrial calcium level in ATCC18804 and 07318 strains was evaluated using Rhod-2 AM staining. The histogram was the quantitative analysis of the percentage of Rhod-2 AM-positive cells, and the data were expressed as mean ± SD. *P < 0.05. [file Image_10.JPEG]

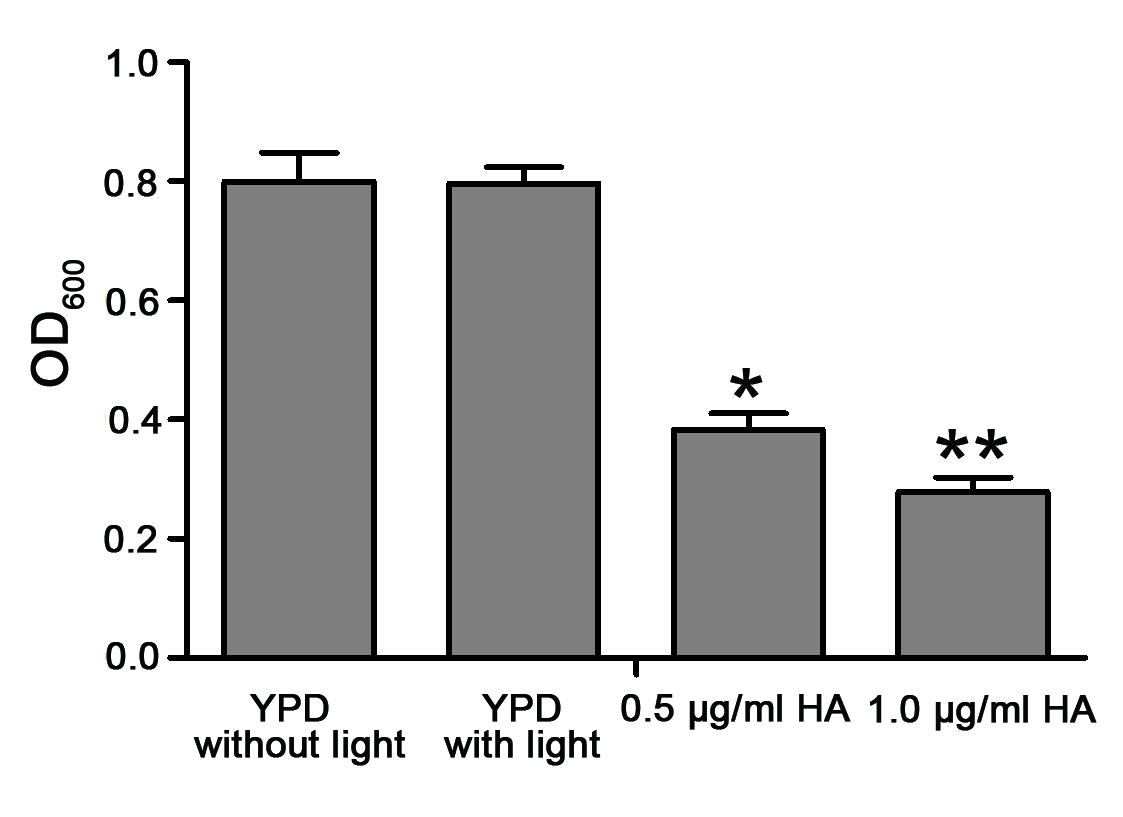

Supplement: FIGURE S11 — Effect of light-only treatment on the growth of C. albicans. *P < 0.05 and ∗∗P < 0.01. [file Image_11.TIF]

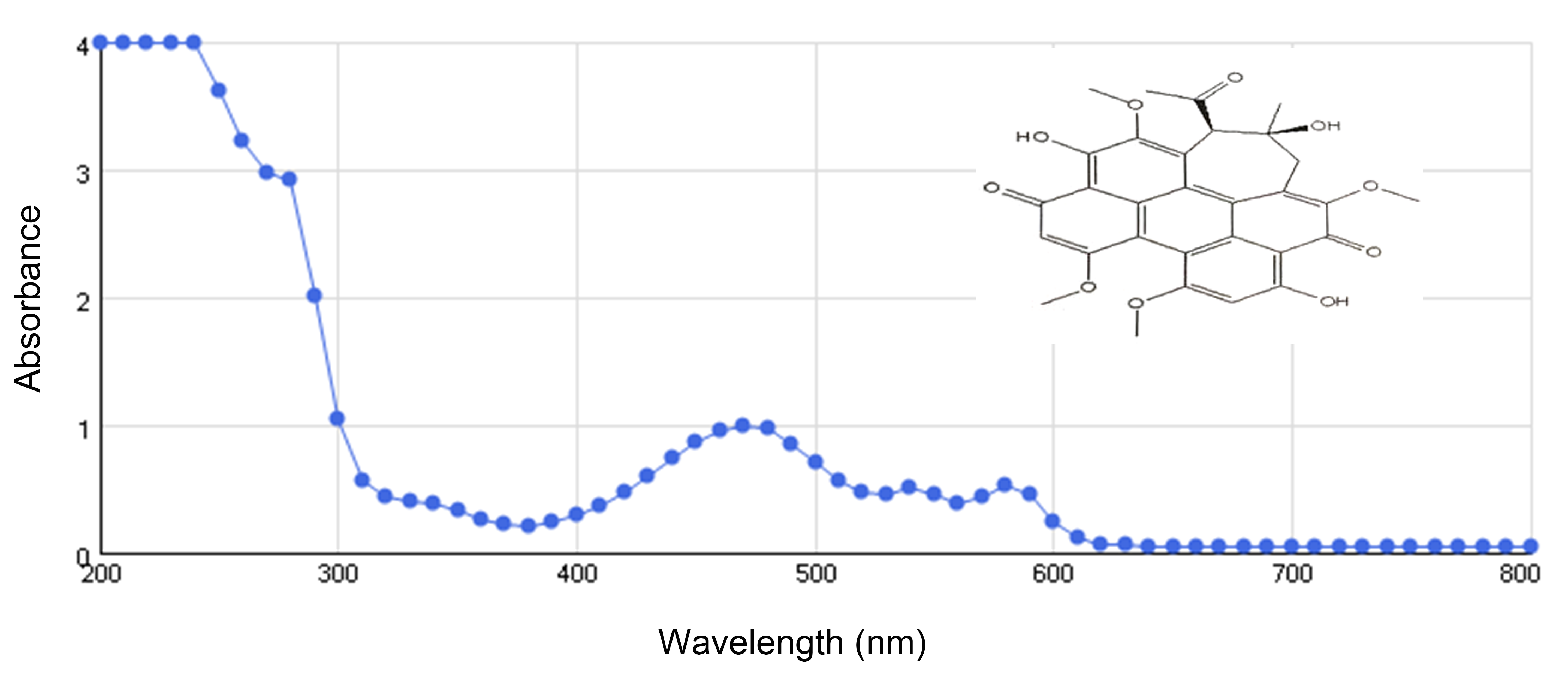

Supplement: FIGURE S12 — The structure and UV-Vis absorption spectrum of HA. [file Image_12.TIF]

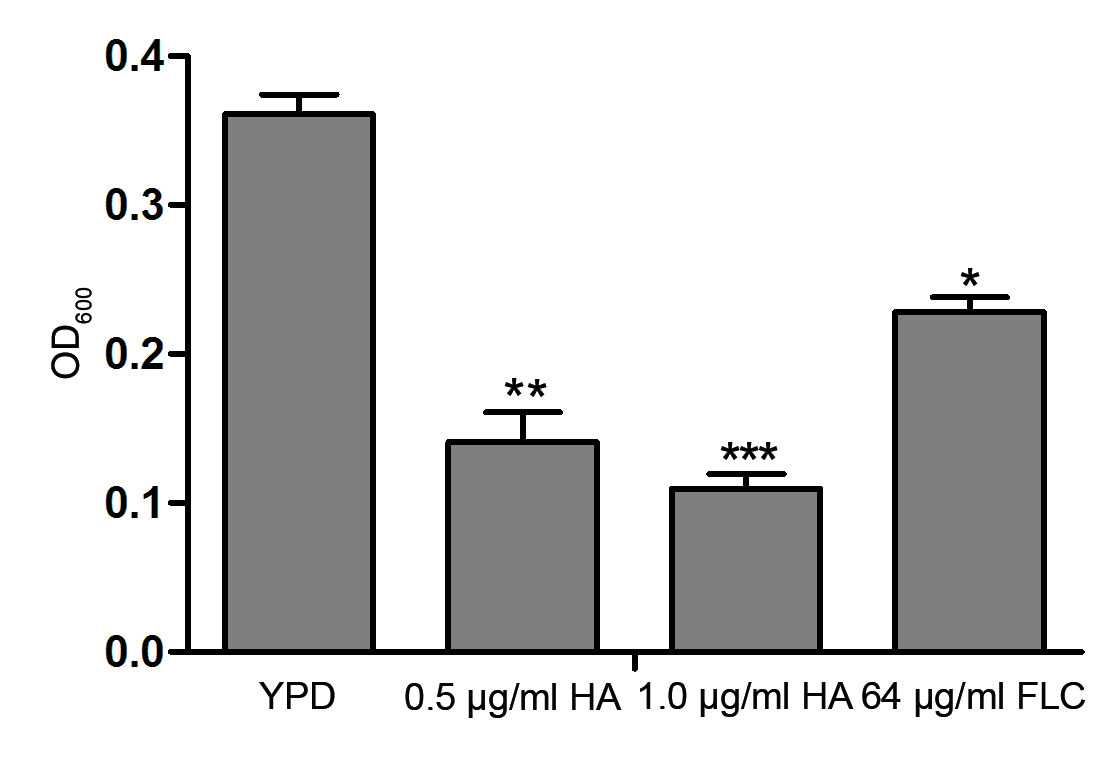

Supplement: FIGURE S13 — Effect of HA and fluconazole (FLC) on C. albicans growth. *P < 0.05, ∗∗P < 0.01, and ∗∗∗P < 0.001. [file Image_13.TIF]

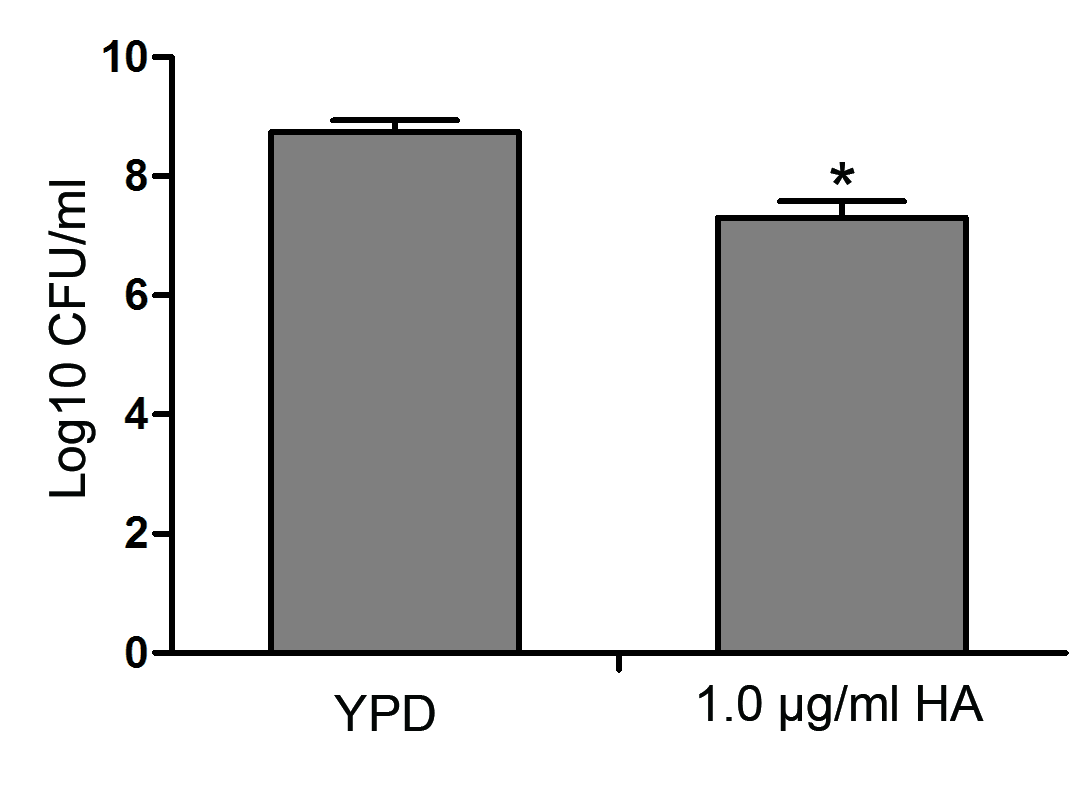

Supplement: FIGURE S14 — Effect of HA treatment on colony-forming units without adjusting to the same number. *P < 0.05. [file Image_14.TIF]

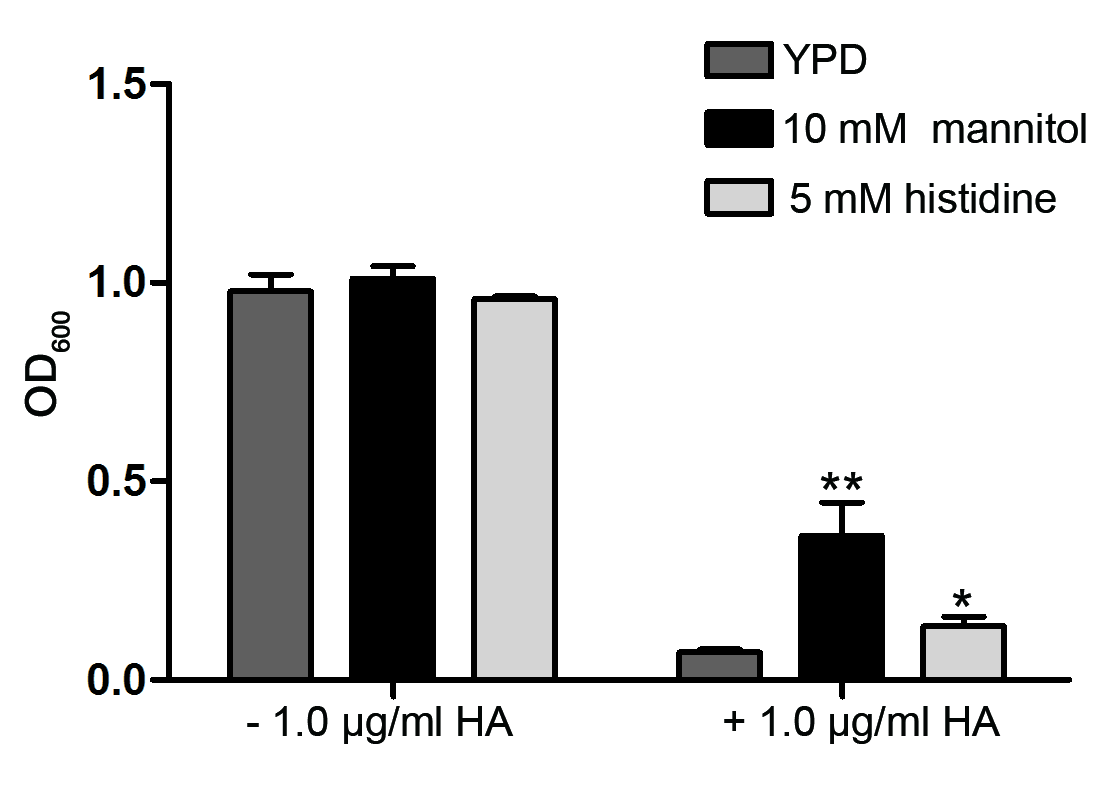

Supplement: FIGURE S15 — Effect of ROS scavengers and quenchers on the photodynamic inactivation of C. albicans by HA. *P < 0.05 and ∗∗P < 0.01. [file Image_15.TIF]
